# circEXOC6B interacting with RRAGB, an mTORC1 activator, inhibits the progression of colorectal cancer by antagonizing the HIF1A-RRAGB-mTORC1 positive feedback loop

**DOI:** 10.1186/s12943-022-01600-1

**Published:** 2022-06-23

**Authors:** Xiaomin Li, Jianjun Wang, Weihao Lin, Qinzi Yuan, Yanxia Lu, Haowei Wang, Yujia Chen, Lixia Chen, Peiling Dai, Huaicheng Long, Xuenong Li

**Affiliations:** 1 Guangdong Province Key Laboratory of Molecular Tumor Pathology, Department of Pathology, Southern Medical University, Guangzhou, 510515 Guangdong Province China; 2 Department of Pathology, Nanfang Hospital, Southern Medical University, Guangzhou, 510515 Guangdong Province China; 3 Department of Histology and Embryology, Wannan Medical College, Wuhu, 241002 Anhui Province China

**Keywords:** Colorectal cancer, circEXOC6B, hsa_circ_0009043, Circular RNA, RRAGB

## Abstract

**Background:**

In recent years, an increasing number of studies have indicated that circular RNA plays crucial roles in regulating tumor development and chemoresistance. Using two high-throughput RNA sequence datasets, we previously found that circEXOC6B was downregulated in colon cancer. However, its role and mechanism in colorectal cancer (CRC) remained unknown.

**Methods:**

Real-time quantitative PCR was used to examine the expression of circEXOC6B in CRC tissues. In vivo and in vitro functional experiments were performed to determine the suppressor role of circEXOC6B in CRC progression. RNA pull-down, mass spectrometry, RNA-binding protein immunoprecipitation, co-immunoprecipitation, fluorescence in situ hybridization, and immunofluorescence were applied to investigate the possible mechanisms connecting circEXOC6B to CRC growth and 5-fluorouracil-induced apoptosis. Chromatin immunoprecipitation, dual-luciferase assay, western blot, and immunohistochemistry were used to explore the mechanisms underlying the HIF1A regulation of RRAGB transcription.

**Results:**

circEXOC6B was downregulated in CRC tissues, and its lower expression was associated with poor prognosis of patients. Functional experiments showed that circEXOC6B inhibited growth and increased the 5-fluorouracil-induced apoptosis of CRC cells in vitro and in vivo. Mechanistically, circEXOC6B inhibited the heterodimer formation of RRAGB by binding to it, thereby suppressing the mTORC1 pathway and HIF1A level. In addition, HIF1A upregulated the transcription of RRAGB by binding to its promoter region. Altogether, the results demonstrated that a HIF1A-RRAGB-mTORC1 positive feedback loop drives tumor progression in CRC, which could be interrupted by circEXOC6B.

**Conclusions:**

circEXOC6B inhibits the progression of CRC and enhances the chemosensitivity of CRC cells to 5-fluorouracil by antagonizing the HIF1A-RRAGB-mTORC1 positive feedback loop. circEXOC6B is a possible therapeutic target for CRC treatment.

**Supplementary Information:**

The online version contains supplementary material available at 10.1186/s12943-022-01600-1.

## Introduction

Colorectal cancer (CRC) is the second most common malignancy and the third leading cause of tumor-related deaths worldwide [1]. The main treatment for CRC is still surgery combined with radiotherapy and chemotherapy. However, the therapeutic efficacy of this approach is strongly limited by an intrinsic or acquired resistance to chemotherapy. Therefore, a crucial step in improving the prognosis of CRC patients is to further explore the comprehensive molecular mechanism underlying CRC development.

Mammalian target of rapamycin complex 1 (mTORC1) is a central regulator of cell growth and survival. It is activated by multiple intracellular and extracellular stimuli, such as growth factors and nutrients [2]. Activated mTORC1 promotes protein and lipid synthesis by phosphorylating the translation initiation factors 4E-binding protein 1 (4E-BP1) and ribosomal protein S6 kinase B1 (S6K) [3, 4]. Dysregulation of mTORC1 signaling is associated with a variety of human diseases, including cancer. For example, mTORC1 signaling is often hyperactive in cancer, which not only promotes continuous tumor growth, but also allows tumor cells to resist chemotherapy [5, 6]. However, the clinical application of mTORC1 inhibitors has achieved limited success due to certain factors, including drug resistance [7], the existence of several feedback loops [8], and tissue toxicity. Ras-related GTP-binding proteins, which include RRAGA, RRAGB, RRAGC, and RRAGD (also known as Rags), are considered the dominant activators of mTORC1 signaling [9]. RRAGA/B combines with RRAGC/D to form heterodimers [9], which recruit mTORC1 to lysosomal membranes to be activated by Rheb-GTP [10, 11]. However, how Rags are regulated remains largely unknown.

We previously found that the circular RNA (circRNA) circEXOC6B (hsa_circ_0009043) was significantly downregulated in colon cancer compared with normal tissue in two high-throughput sequencing datasets [12, 13]. However, the role and mechanism of circEXOC6B in CRC remained unknown. In the present study, we found that circEXOC6B can interfere with the heterodimer formation of RRAGB and RRAGC/D by competitively binding to RRAGB, thereby blocking the mTORC1 pathway from perceiving growth signals and leading to lower levels of hypoxia-inducible factor 1 alpha (HIF1A). Interestingly, we determined that HIF1A could bind to the promoter region of RRAGB to promote its transcription. Hence, a HIF1A-RRAGB-mTORC1 positive feedback loop drives CRC progression. However, circEXOC6B blocked this positive feedback loop, as mentioned above, thereby inhibiting CRC growth and increasing the sensitivity of tumors to 5-fluorouracil (5-FU) chemotherapy. Our research provides an experimental foundation for CRC treatment that acts by preventing cancer cells from receiving growth-promoting signals and suggests circEXOC6B as a potential target for CRC treatment.

## Methods

### Cell culture

Human CRC cell lines SW480, SW620, RKO, Caco-2, HCT116, LoVo and HT29 were purchased from the American Type Culture Collection (ATCC, Manassas, VA, USA). Cells were cultured in RPMI 1640 medium (#C11875500BT, Gibco, Grand Island, NY, USA) containing 10% fetal bovine serum at 37 °C in a humidified atmosphere with 5% CO_2_.

### CRC tissues

The 78 paired of CRC tissues and adjacent noncancerous tissues were collected from Nanfang Hospital, Southern Medical University (Guangzhou, China). The consents were obtained from all patients and the use of clinical materials for research purposes were approved by the Ethics Committee of Southern Medical University (Guangzhou, China). CRC tissues were taken from the general surgically resected CRC specimens, and the matched adjacent non-cancerous tissues were taken from the site around 5–10 cm away from the edge of the tumors. These specimens were immediately frozen in liquid nitrogen until use. All samples were diagnosed as adenocarcinoma by pathologists. All patients were not received preoperative chemoradiotherapy.

The cDNA microarray of colon cancer tissues (HColA095Su02) was purchased from Shanghai Outdo Biotech Company. It contains 80 colon cancer tissues and 15 adjacent noncancerous tissues along with each patient’s gender, age, clinical stage, lymphatic metastasis status and survival information, that were recorded and archived in the National Engineering Center for Biochip. The use of cDNA microarray for research purposes was approved by the Ethics Committee of Shanghai Outdo Biotech Company.

### RNA extraction, reverse transcription, polymerase chain reaction (PCR), sanger sequencing, real time quantitative PCR (RT-qPCR), RNase R digestion, actinomycin D treatment, separation of cytoplasmic and nuclear fractions, CCK8, colony formation and subcutaneous tumor model

These assays were performed as previous described [14, 15]. The comparative C(T) method was used for RT-qPCR data analysis [16]. 18sRNA, β-actin and GAPDH were used as the internal reference in RT-qPCR assay. All primers used in the research were synthesized by Ruibiotech (Guangzhou, China) (Table S1). Three to Four weeks old, female BALB/C nude mice were purchased from Guangdong Medical Experimental Animal Center and all animal experimental protocols were approved by the Animal Care and Use Committee of Southern Medical University.

### Small interfering RNA (siRNA), lentivirus, vector construction and transfection

All siRNAs were designed and synthesized by GenePharma (Suzhou, China) or RiboBio (Guangzhou, China) (Table S2). sh-circEXOC6B lentivirus vector was constructed and packaged into lentivirus particles by Genechem (Shanghai, China). All vectors used in the study were constructed by Ruibiotech (Guangzhou, China). The plasmid pCD5-ciR (Geneseed Biotech, Guangzhou, China) was used to construct circEXOC6B overexpression vector. CRC cells were transfected with siRNAs or vectors using lipofectamine3000 (#L3000015, Invitrogen, USA) according to the instruction.

### 5-fluorouracil (5-FU) treatment in vitro and in vivo

SW620 and HCT116 Cells were treated with 5 μg/ml and 1 μg/ml 5-FU (H31020593, Shanghai Xudong Haipu Pharmaceutical Co., Ltd., China) for 24 h, then followed by CCK8, flow cytometry, TUNEL and western blot detection. For in vivo experiment, we established subcutaneous tumor models using HCT116 with stable knockdown of circEXOC6B or control cells. When the subcutaneous tumors grew to 0.5 cm, nude mice were intraperitoneally injected with 20 μg 5-FU per gram of body weight, while the control group was injected with saline, 3 times a week, for 2 weeks.

### EdU cell proliferation detection

EdU Cell Proliferation Kit with Alexa Fluor 555 (CX003, Epizyme, Shanghai, China) was used to detect the proliferation of cells according to the instruction. In short, 1 × EdU working solution was added into cells and incubated for 2 h. Then, cells were fixed, washed and incubated with the reaction solution at room temperature for 30 min. DAPI stained nucleus. The number of positive cells was counted and calculated.

### Fluorescence in situ hybridization (FISH) and immunofluorescence (IF)

Fluorescent in Situ Hybridization Kit (C10910, RiboBio, Guangzhou, China) was used to perform FISH on CRC cells according to the instruction and our previous study [14]. In brief, CRC cells were fixed, washed, permeabilized and followed by hybridization reaction with Cy3-labeled single stranded DNA oligo probes against circEXOC6B (RiboBio, Guangzhou, China). For paraffin sections, dewaxing and digestion were required prior to FISH. To confirm the co-localization of circEXOC6B and RRAGB in CRC cells, IF was performed following FISH assay. The anti-flag antibody (YM3001, Immunoway, USA) and alexa fluor 488 labeled goat anti-mouse IgG (ZF-0512, ZSGB-BIO, China) were used in IF assay. Images were obtained under a confocal microscope.

### RNA pull-down, mass spectrometry (MS) and RNA binding protein immunoprecipitation (RIP)

These experiments were performed as previous described [17]. Briefly, proteins binding to circEXOC6B were pulled down and separated by SDS-PAGE electrophoresis followed by silver staining (#P00175, Fast Silver Stain Kit, Beyotime, Shanghai, China). Then, the differential protein band was cut off and identified by MS (BGI, Shenzhen, China). The anti-flag antibody (YM3001, Immunoway, USA) and mouse control IgG (AC011, ABclonal, China) were used in RIP assay. Western blot was used to verify the enrichment of RRAGB (1:1000, 13,023–1-AP, Proteintech, USA). PCR were used to determine the enrichment of circEXOC6B.

### Coimmunoprecipitation (co-IP)

In short, the primary anti-flag antibody (YM3001, Immunoway, USA) or mouse control IgG (AC011, ABclonal, China) were added into cell lysate and the mixture was incubated at 4 °C for 1 h. Thereafter, 20 μl A/G plus-Agarose beads (SC-2003, SANTA Cruz Biotechnology, USA) were added and incubated at 4 °C overnight. The proteins were eluded and detected using western blot with the primary anti-flag (YM3001, Immunoway, USA), anti-RRAGC (YT3992, Immunoway, USA), anti-RRAGD (YN1164, Immunoway, USA) and the secondary antibody (M21008, 1:1000 dilution, Abmart, China).

### Western blot

The total protein was separated by SDS-PAGE electrophoresis and transferred onto PVDF membrane (#IPVH00010, Millipore, USA). Then, the PVDF membrane was incubated with the following primary antibodies at 4 °C overnight: anti-RRAGB (1:1000, 13,023–1-AP, Proteintech, USA), anti-mTOR (1:500, 66,888–1-IG, Proteintech, USA), anti-p-mTOR (Ser2448) (1:1000, YP0176, Immunoway, USA), anti-S6K (1:1000, YT3555, Immunoway, USA), anti-p-S6K (Thr389) (1:1000, YP1427, Immunoway, USA), anti-HIF1A (1:1000, YT2133, Immunoway, USA), anti-pro-caspase3 (1:1000, AB32150, Abcam, USA), anti-cleaved-caspase3 (1:500, YM3431, Immunoway, USA), anti-PARP (1:1000, AB191217, Abcam, USA), anti-cleaved-PARP (1:1000, AB32064, Abcam, USA), and anti-β-tubulin (1:2000, 10,094–1-AP, Proteintech, USA). Subsequently, the PVDF membrane was incubated with the corresponding HRP labeled goat anti-mouse (1:500, BS12478, Bioworld, USA) or goat anti-rabbit IgG (1:500, BS13278, Bioworld, USA) at room temperature for 1 h. FDBio-Dura ECL Kit (FD8020, FDbio, China) was used to visualize the protein bands.

### Immunohistochemistry (IHC)

IHC staining and H-Score calculating were performed as our previous research [17]. The primary antibodies used in the assay were anti-RRAGB (1:300, 13,023–1-AP, Proteintech, USA), anti-p-mTOR (Ser2448) (1:200, YP0176, Immunoway, USA), anti-HIF1A (1:300, YT2133, Immunoway, USA), and anti-cleaved-caspase3 (1:100, YM3431, Immunoway, USA).

### Chromatin immunoprecipitation (ChIP) and dual-luciferase reporter assay

The SimpleChIP® Enzymatic Chromatin IP Kit (#9003, Cell Signaling Technology, USA) was utilized to perform ChIP assay according to the specification. In brief, CRC cells were fixed, collected and lysed. Then, pelleted nuclei by centrifugation followed by DNA digestion. This digested chromatin was incubated with HIF1A antibody (1:200 dilution, YT2133, Immunoway, USA) and magnetic beads to perform immunoprecipitation. The chromatin was eluted from the magnetic beads and detected using PCR assay. The Dual Luciferase Reporter Assay System (E1910, Promega, WI, USA) was used to perform dual-luciferase reporter assay according to the instruction and previous research [14]. HIF1A siRNA and HIF1A activator ML228 (5 μM, HY-12754, MCE, China) were used in the dual-luciferase reporter assay.

### Flow cytometry and TUNEL

Cell Cycle Detection Kit (KGA512, KeyGen Biotech, China) was used to detect the cell cycle progression of CRC cells according to the instruction. Annexin V-APC/PI Apoptosis Detection Kit (KGA1030, KeyGen Biotech, China) and One-step TUNEL cell apoptosis assay kit (red TRITC labeled fluorescence) (KGA7061, KeyGen Biotech, China) were used to detect the apoptosis according to the instructions.

### Statistical analyses

Statistical analysis was performed with GraphPad Prism6.0 (GraphPad Software, La Jolla, CA, USA) and SPSS23.0 software (IBM, USA). The measurement data are presented as mean ± standard deviation. A two-tailed Student’s t-test was used to detect the difference between two groups. The comparison of circEXOC6B expression in CRC tissues and matched noncancerous tissues was analyzed using the paired t-test. The relationships between circEXOC6B expression and clinicopathological characteristics were determined by χ2 test. Kaplan-Meier method and Log rank test were used to evaluate the difference of survival time between low and high circEXOC6B expression groups. Univariate and multivariate Cox proportional hazards model were performed to evaluate the prognostic value of circEXOC6B. Pearson’s correlation coefficient was used for correlation analysis of RRAGB and HIF1A expression. Differences were considered to be significant at *P < 0.05, **P < 0.01, ***P < 0.001, ****P < 0.0001. NS: not significant.

## Results

### circEXOC6B is down regulated in CRC tissue

Differentially expressed circRNAs between colon cancer and normal tissue were screened by high-throughput sequencing (PMID25624062) [12] and circEXOC6B was found to be the second most downregulated circRNA in colon cancer (Fig. S1A). Consistently, another high-throughput sequencing dataset (GSE77661) from the GEO database also demonstrated reduced circEXOC6B expression in colon cancer [13] (Fig. S1B). circBase (http://circbase.org/) showed that circEXOC6B is generated from exons 3–6 of its parental gene EXOC6B and that its mature length is 390 bp. We performed a series of experiments to verify that circEXOC6B is indeed a circRNA originating from EXOC6B. First, we designed two pairs of divergent primers (1 and 2), which could amplify 100 bp and nearly the full-length of circEXOC6B, respectively (Fig. S1C). Then, we used primer 2 to amplify circEXOC6B to detect the junction sequences of circEXOC6B by Sanger sequencing. The results verified that all junction sequences between the exons of circEXOC6B were correct (Fig. S1C). In addition, circEXOC6B could only be amplified from cDNA in CRC cells using the divergent primer, and not from genomic DNA, which supported the formation of circEXOC6B by post-transcriptional back-splicing (Fig. S1D). Finally, RNase R or actinomycin D treatment demonstrated that circEXOC6B was more stable than linear GAPDH mRNA (Fig. S1E and F), which indicated that circEXOC6B possesses a closed loop structure without 5′ or 3′ terminals.

**Fig. 1:**
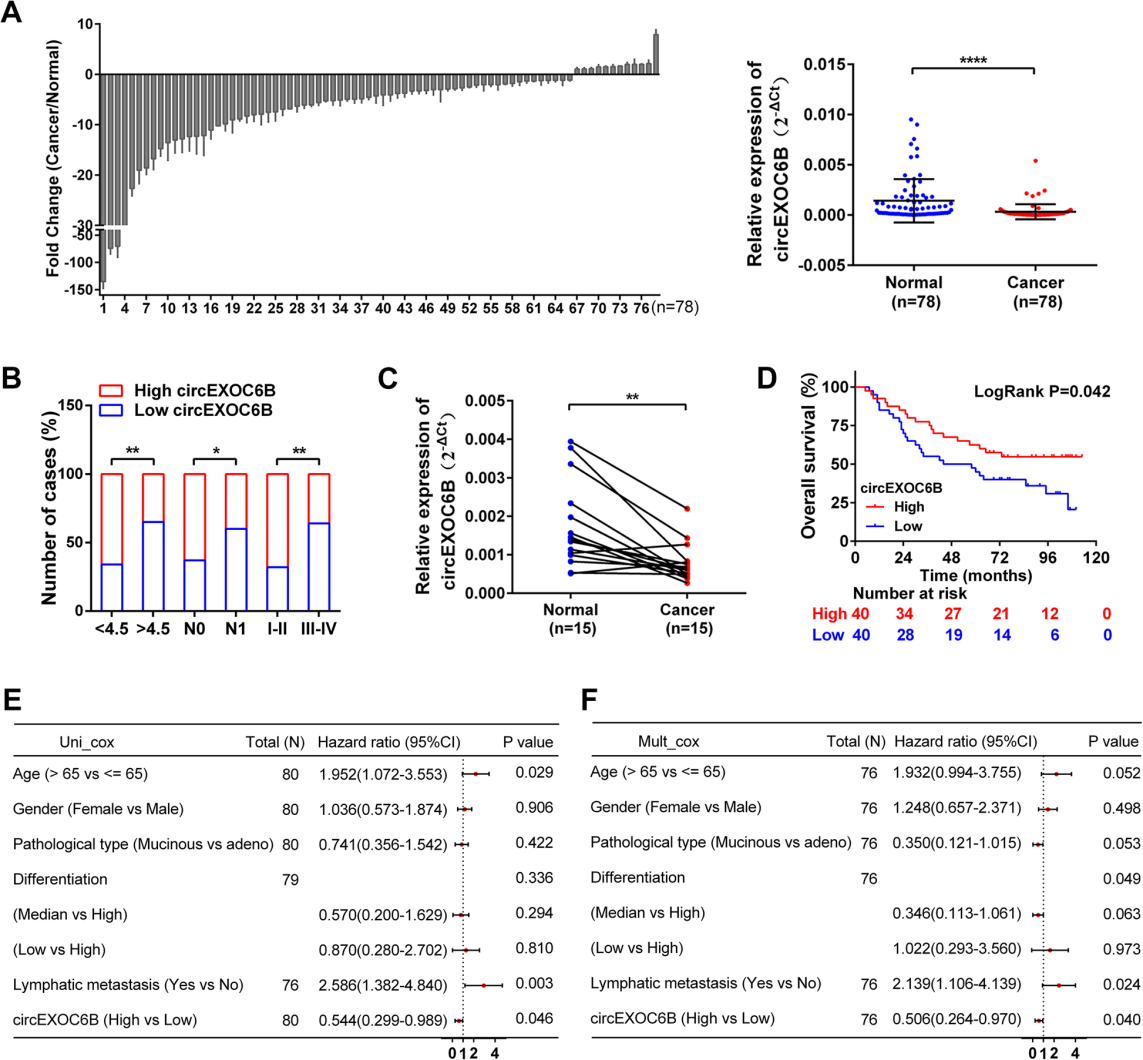
circEXOC6B is downregulated in CRC tissue. **A** The relative expression levels of circEXOC6B in CRC tissues (Cancer, *n* = 78) and the adjacent noncancerous tissues (Normal, *n* = 78) were determined by RT-qPCR. **B** The expression of circEXOC6B was negatively correlated with tumor size, lymphatic metastasis, and TNM stage. **C** RT-qPCR showed the expression of circEXOC6B was lower in 15 cases of cancer tissues than in the matched adjacent tissues in the colon cancer cDNA microarray. **D** Kaplan-Meier survival analysis revealed that the patients with high circEXOC6B expression had a higher OS rate than patients with low circEXOC6B expression. **E **and** F** Forest plots of the univariate and multivariate Cox proportional hazards model verified the prognostic value of circEXOC6B

We further determined circEXOC6B expression in CRC tissues using RT-qPCR. As shown in Fig. 1A, circEXOC6B was downregulated in 78 cases of CRC tissues (Cancer) compared with the adjacent noncancerous tissues (Normal). To explore the relationship between circEXOC6B expression and the clinicopathological parameters of patients with CRC, we divided the 78 CRC patients into a high expression group (*n* = 39) and a low expression group (*n* = 39) according to the median expression of circEXOC6B. Statistical analysis revealed that the expression of circEXOC6B was negatively correlated with tumor size, lymphatic metastasis, and TNM stage (Fig. 1B and Table S3). In addition, we also detected the expression of circEXOC6B in a colon cancer cDNA microarray, which contained 80 cases of cancer tissues and 15 cases of adjacent tissues. RT-qPCR results showed that circEXOC6B expression was also lower in cancer tissues than in the matched adjacent tissues (Fig. 1C). Moreover, Kaplan-Meier survival analysis revealed that patients with high circEXOC6B expression had a higher overall survival (OS) rate (54.8%) than patients with low circEXOC6B expression (20.6%) (*P* = 0.042) (Fig. 1D). Next, a univariate Cox proportional hazards model showed that patients with higher circEXOC6B expression had a lower risk of death (hazard ratio [HR] = 0.544, 95% CI = 0.299–0.989, *P* = 0.046) than patients with lower circEXOC6B expression (Fig. 1E). Finally, multivariate regression analysis further confirmed that high circEXOC6B expression reduced the risk of death by 49.4% (HR = 0.506, 95% CI = 0.264–0.970, *P* = 0.040) (Fig. 1F). These results demonstrated that circEXOC6B was associated with improved OS and was an independent prognostic factor in patients.

### circEXOC6B inhibits the growth of CRC cells in vitro and in vivo

**Fig. 2:**
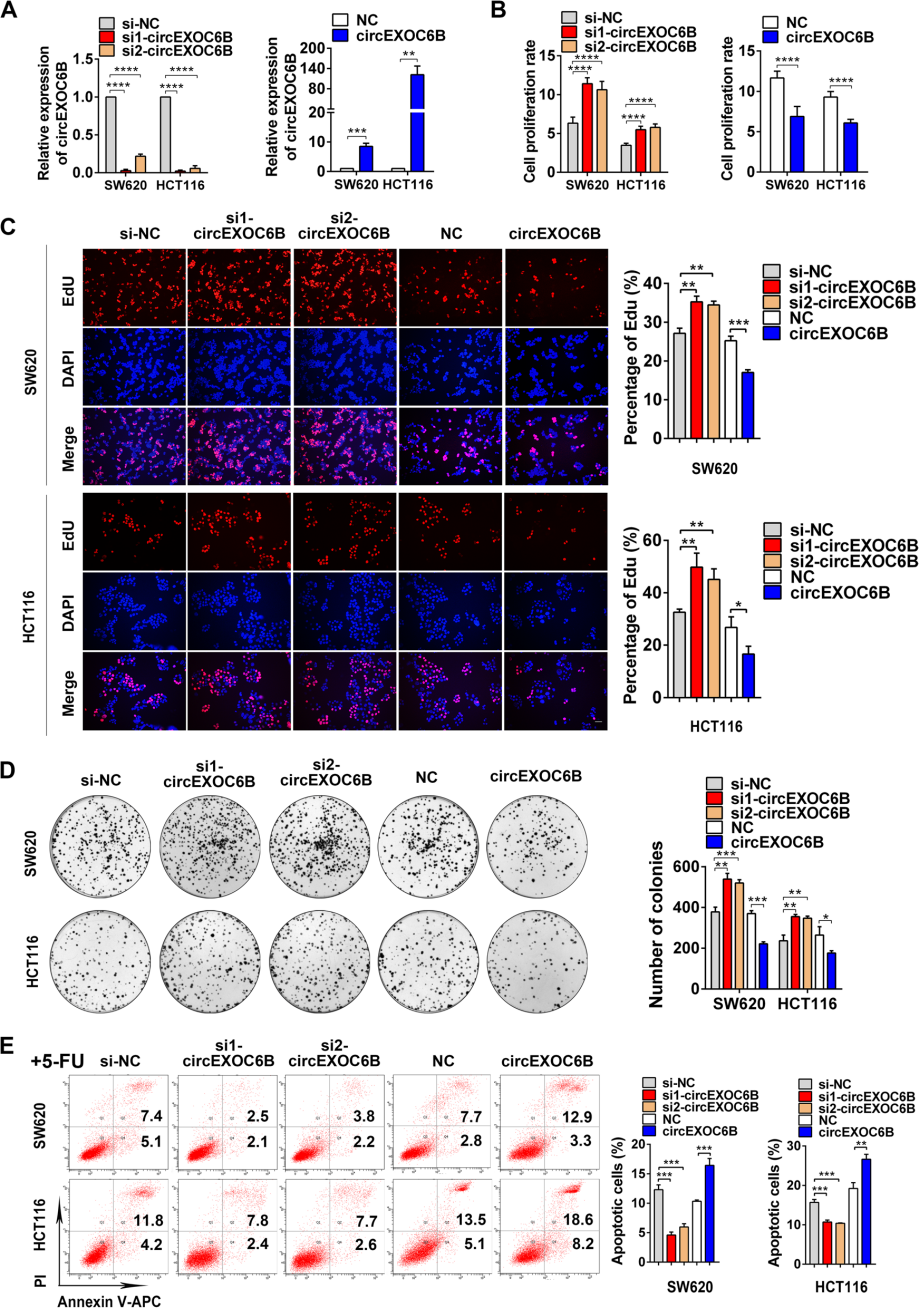
circEXOC6B inhibits the growth of CRC cells in vitro and in vivo. **A** The expression of circEXOC6B in SW620 and HCT116 after transfected with siRNAs or a circEXOC6B overexpression vector. **B** CCK8 and **C** EdU assays indicated that the lower circEXOC6B levels promoted the proliferation of CRC cells while the higher circEXOC6B levels inhibited the proliferation. Scale: 50 μm. **D** Colony formation assay demonstrated that knockdown of circEXOC6B expression promoted the clonogenicity of SW620 and HCT116 while overexpression of circEXOC6B suppressed cancer cell clonogenicity. **E** Flow cytometry showed that the decreased circEXOC6B levels reduced the 5-FU-induced apoptosis of CRC cells, whereas the elevated circEXOC6B levels had the opposite effect

To investigate the role of circEXOC6B in CRC progression, we knocked down circEXOC6B expression in CRC cells using siRNAs specifically targeting the splicing junction of circEXOC6B and upregulated circEXOC6B expression using a circEXOC6B overexpression vector (Fig. 2A and S2A). Flow cytometry and CCK8, EdU, and colony formation assays showed that lower circEXOC6B levels significantly promoted the proliferation, clonogenicity, and cell cycle progression of CRC cells while the higher circEXOC6B levels suppressed cancer cell viability and clonogenicity (Figs. 2B–D and S2B–E). In addition, flow cytometry indicated that the decreased circEXOC6B levels inhibited the 5-FU-induced apoptosis of CRC cells, whereas the elevated circEXOC6B levels had the opposite effect (Fig. 2E).

Furthermore, using a lentiviral approach, we stably silenced circEXOC6B expression in SW620 and HCT116 cells for further functional research (Fig. S3A). Subsequent CCK8 and colony formation assays showed that the stable depletion of circEXOC6B boosted the proliferation and clonogenicity of CRC cells compared with the control cells (Fig. S3B, C). Consistent with the in vitro functional experiments, subcutaneous tumor models demonstrated that the stable downregulation of circEXOC6B accelerated the growth of CRC cells in vivo (Fig. S3D, E).

### circEXOC6B binds to RRAGB to interfere with its heterodimer formation

**Fig. 3:**
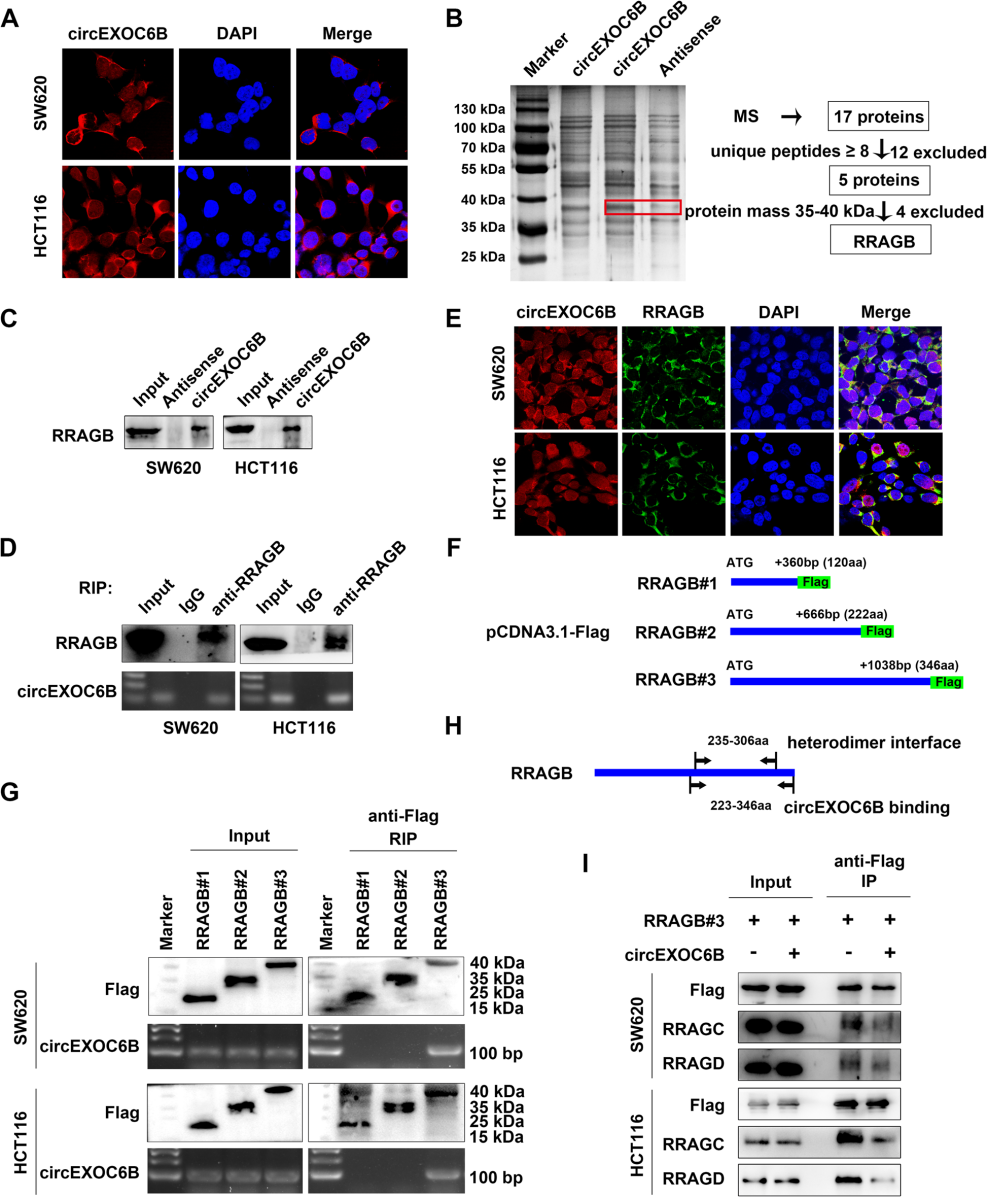
circEXOC6B binds to RRAGB to interfere with its heterodimer formation. **A** FISH showed that circEXOC6B was mainly resided in the cytoplasm of CRC cells; Red: circEXOC6B, Blue: nuclei. **B** RNA pull-down and mass spectrometry (MS) were performed to identify proteins binding to circEXOC6B. The red frame marked RRAGB. **C** RNA pull-down followed by western blot confirmed the binding of circEXOC6B and RRAGB. **D** RIP assay indicated that RRAGB could enrich circEXOC6B. **E** FISH and IF labeled circEXOC6B and RRAGB in CRC cells. **F** RRAGB expression vectors of different lengths (#1–3) were constructed using pCDNA3.1-Flag vector. **G** RIP assay showed that the 223–346-aa region of RRAGB was responsible for the interaction with circEXOC6B. **H** The 223–346-aa region of RRAGB contains the binding site for the heterodimerization of RRAGB with RRAGC/D (235–306-aa, NCBI gene database). **I** Co-IP demonstrated that the binding of RRAGC and RRAGD to RRAGB decreased with circEXOC6B overexpression

To explore the molecular mechanisms underlying the circEXOC6B regulation of CRC progression, we first determined the subcellular localization of circEXOC6B in CRC cell lines and CRC clinical samples using FISH, PCR, and RT-qPCR. The results indicated that circEXOC6B mainly resided in the cytoplasm of CRC cells (Figs. 3A and S4). circRNAs in the cytoplasm can interact with miRNAs or proteins, and their binding to protein was believed to be their dominant function, although it remains poorly elucidated. To identify the proteins interacting with circEXOC6B, we performed RNA pull-down in SW620 and HCT116 cells and observed a differential band between 35 and 40 kDa (Figs. 3B and S5). Subsequently, 17 proteins were identified by mass spectrometry. Of these, 12 were excluded because their unique peptides numbered less than 8. In the remaining five proteins, RRAGB was the only protein with a molecular mass between 35 and 40 kDa (Fig. 3B and Table S4). Therefore, RRAGB was the candidate protein binding to circEXOC6B. RNA pull-down followed by western blot confirmed the binding of circEXOC6B and RRAGB (Fig. 3C). Furthermore, RIP assay verified that RRAGB could enrich circEXOC6B (Fig. 3D). To further confirm the interaction of circEXOC6B and RRAGB, we labeled circEXOC6B and RRAGB using FISH and IF in CRC cells. The results revealed the colocalization of circEXOC6B and RRAGB in the cytoplasm of CRC cells (Fig. 3E).

Moreover, using the catRAPID database [18], we predicted the possible binding sites of RRAGB with circEXOC6B. The results showed that the most probable region for circEXOC6B binding in RRAGB was located at around the 250-aa site (Fig. S6). Thus, we constructed RRAGB expression vectors of different lengths (#1–3) (Fig. 3F). RIP assay revealed that the 223–346-aa region of RRAGB was responsible for the interaction with circEXOC6B (Fig. 3G). Interestingly, this region contains the binding site for the heterodimerization of RRAGB with RRAGC/D (235–306-aa, NCBI gene database) (Fig. 3H). Hence, we speculated that circEXOC6B might competitively interfere with the formation of RRAGB–RRAGC/D heterodimers. To test this hypothesis, we performed a Co-IP assay in CRC cells after their transfection with flag-labeled RRAGB expression vector. Western blotting showed that the binding of RRAGC and RRAGD to RRAGB decreased with circEXOC6B overexpression (Fig. 3I). These results indicated that circEXOC6B can bind to RRAGB and inhibit the heterodimer formation of RRAGB and RRAGC/D.

### circEXOC6B suppresses the mTORC1 pathway

**Fig. 4:**
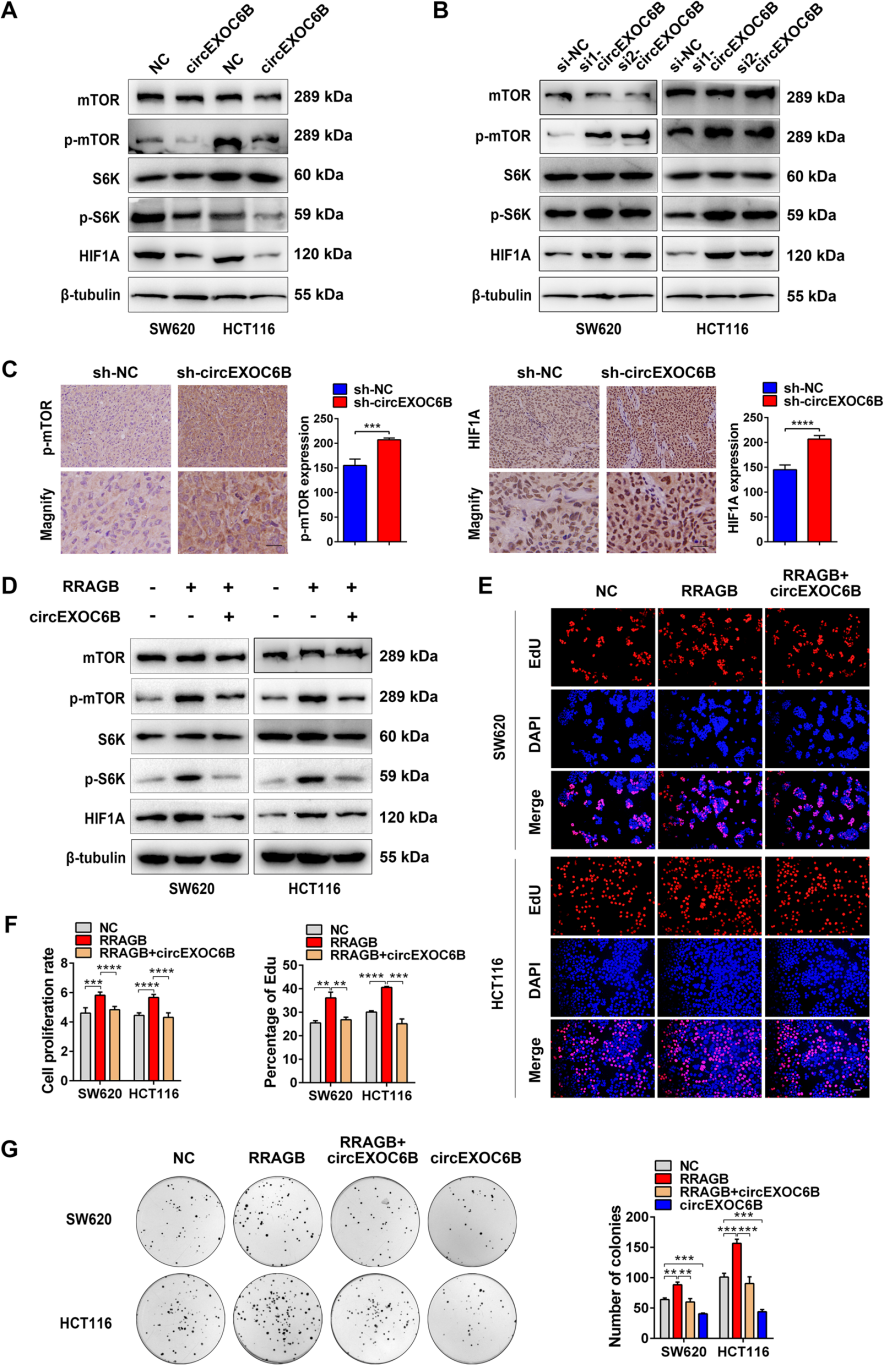
circEXOC6B suppresses the mTORC1 pathway. **A** Western blot results showed that overexpression of circEXOC6B suppressed the mTORC1 pathway. **B** Knockdown of circEXOC6B activated the mTORC1 pathway. **C** IHC indicated that p-mTOR and HIF1A expression was upregulated in the subcutaneous tumors with stable silencing of circEXOC6B expression. Scale: 20 μm. **D** Western blot results showed that the p-mTOR, p-S6K, and HIF1A elevation caused by RRAGB could be reversed by co-transfection with circEXOC6B. **E** EdU, **F** CCK8, **G** Colony formation assays showed that the enhanced proliferation and clonogenicity of CRC cells induced by RRAGB could be rescued by overexpression of circEXOC6B. Scale: 50 μm

Given that formation of the RRAGB-RRAGC/D heterodimer is required for activation of the mTORC1 pathway, we deduced that circEXOC6B might suppress the mTORC1 pathway by interfering with heterodimer formation. Western blot results showed that overexpression of circEXOC6B reduced the expression of p-mTOR, p-S6K, and HIF1A while its downregulation increased their expression (Fig. 4A and B). Consistent with the western blot results, IHC indicated that p-mTOR and HIF1A expression was also upregulated in the subcutaneous tumors with stable silencing of circEXOC6B expression (Fig. 4C).

In addition, we performed rescue assays to further verify the involvement of RRAGB in the regulation of circEXOC6B in the mTORC1 pathway. Western blotting showed that the p-mTOR, p-S6K, and HIF1A elevation caused by RRAGB could be reversed by co-transfection with circEXOC6B (Fig. 4D). Similarly, the enhanced proliferation and clonogenicity of CRC cells induced by RRAGB could also be rescued by overexpression of circEXOC6B (Fig. 4E - G). These results support the hypothesis that circEXOC6B inhibits the mTORC1 pathway to promote CRC growth by competitively binding to RRAGB.

### CRC has a HIF1A-RRAGB-mTORC1 positive feedback loop that is antagonized by circEXOC6B

**Fig. 5:**
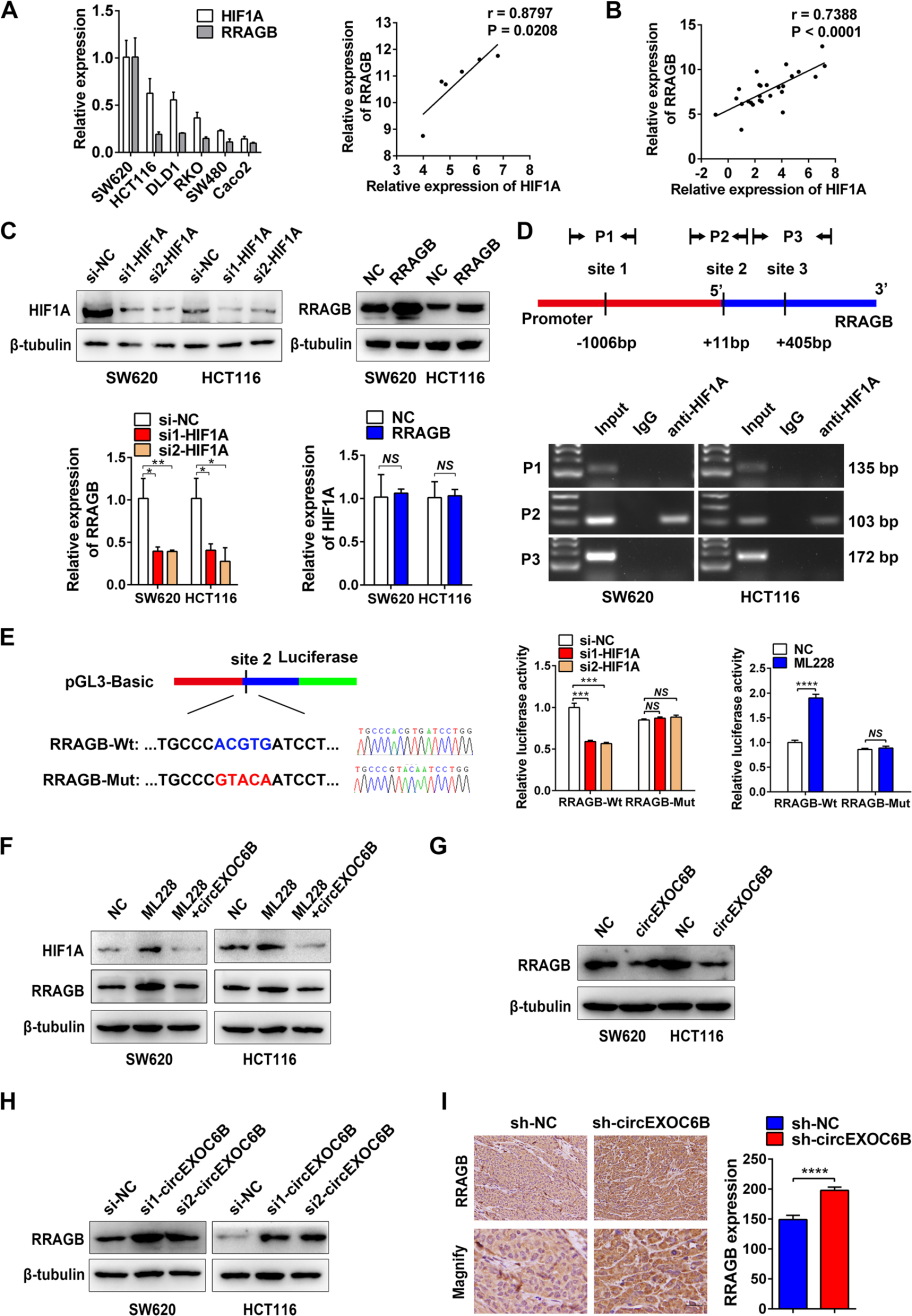
CRC has a HIF1A-RRAGB-mTORC1 positive feedback loop that is antagonized by circEXOC6B. **A** RT-qPCR showed a positive relationship between HIF1A and RRAGB expression in six CRC cell lines. **B** RT-qPCR indicated the expression levels of HIF1A and RRAGB were positively correlated in 26 CRC tissues. **C** RT-qPCR was performed to detect the expression of RRAGB and HIF1A mRNA after knockdown of HIF1A or overexpression of RRAGB in SW620 and HCT116. **D** The promoter region of RRAGB contained three HREs and ChIP assay indicated that HIF1A bound to the second HRE site of the promoter. **E** Dual-luciferase reporter assay confirmed that HIF1A bound to the second HRE site of RRAGB promoter and promoted luciferase transcription. Wt: wild HRE, Mut: mutant HRE. **F** HIF1A activator ML228 was used to upregulate HIF1A expression, and western blotting showed that the RRAGB level was increased. However, the effect could be abrogated by circEXOC6B. **G** Overexpression of circEXOC6B downregulated RRAGB expression. **H** Knockdown of circEXOC6B elevated RRAGB expression. **I** IHC showed that subcutaneous tumors with stable silencing of circEXOC6B expression had higher expression of RRAGB than the control group. Scale: 20 μm

When we determined the expression of HIF1A and RRAGB in six CRC cell lines using RT-qPCR, we surprisingly found that there was a positive relationship between HIF1A and RRAGB expression (Fig. 5A). Moreover, the expression levels of HIF1A and RRAGB were also positively correlated in 26 CRC tissues (Fig. 5B). In addition, their positive correlation in CRC was further validated using GEO (Fig. S7A) and The Cancer Genome Atlas (TCGA) (Fig. S7B) datasets. These findings motivated us to explore whether there is transcriptional regulation between HIF1A and RRAGB expression. RT-qPCR revealed that knockdown of HIF1A caused the downregulation of RRAGB mRNA expression, whereas overexpression of RRAGB had no effect on HIF1A mRNA expression (Fig. 5C). Furthermore, we found that the promoter region of RRAGB contained three HREs (RCGTG), which are the binding sites for the transcription factor HIF1A (Fig. 5D). Subsequent ChIP indicated that HIF1A bound to the second HRE site of the promoter (Fig. 5D). For further confirmation, we constructed RRAGB-Wt and RRAGB-Mut (mutant HRE) luciferase vectors to perform a dual-luciferase reporter assay (Fig. 5E). The results showed that luciferase activity was reduced after knockdown of HIF1A expression but increased after overexpression of HIF1A using the HIF1A activator ML228 (5 μM) (Fig. 5E). These results suggest that HIF1A can bind to the promoter of RRAGB and promote RRAGB transcription.

The mTORC1 pathway enhances the translation of HIF1A. Therefore, a HIF1A-RRAGB-mTORC1 positive feedback loop exists in CRC to drive tumor progression, and circEXOC6B might antagonize the loop by binding to RRAGB. We used the HIF1A activator ML228 to upregulate HIF1A, and western blotting showed that the RRAGB level was increased. However, circEXOC6B was able to abrogate the effect and to decrease the expression of RRAGB and HIF1A (Fig. 5F). In addition, overexpression of circEXOC6B downregulated RRAGB expression while knockdown of circEXOC6B elevated RRAGB expression (Fig. 5G and H). In line with the western blot results, IHC showed that subcutaneous tumors with stable silencing of circEXOC6B expression had higher expression of RRAGB than the control group (Fig. 5I). To further verify the inhibitory role of circEXOC6B in the positive feedback loop, we used IHC to evaluate the expression of HIF1A, RRAGB, and p-mTOR in the 22 CRC clinical samples used in Table S3, including 11 cases of low circEXOC6B expression and 11 cases of high circEXOC6B expression. The results showed that HIF1A, RRAGB, and p-mTOR levels were lower in the high circEXOC6B group than in the low circEXOC6B group (Fig. S8).

### circEXOC6B enhances the sensitivity of CRC cells to 5-FU

**Fig. 6:**
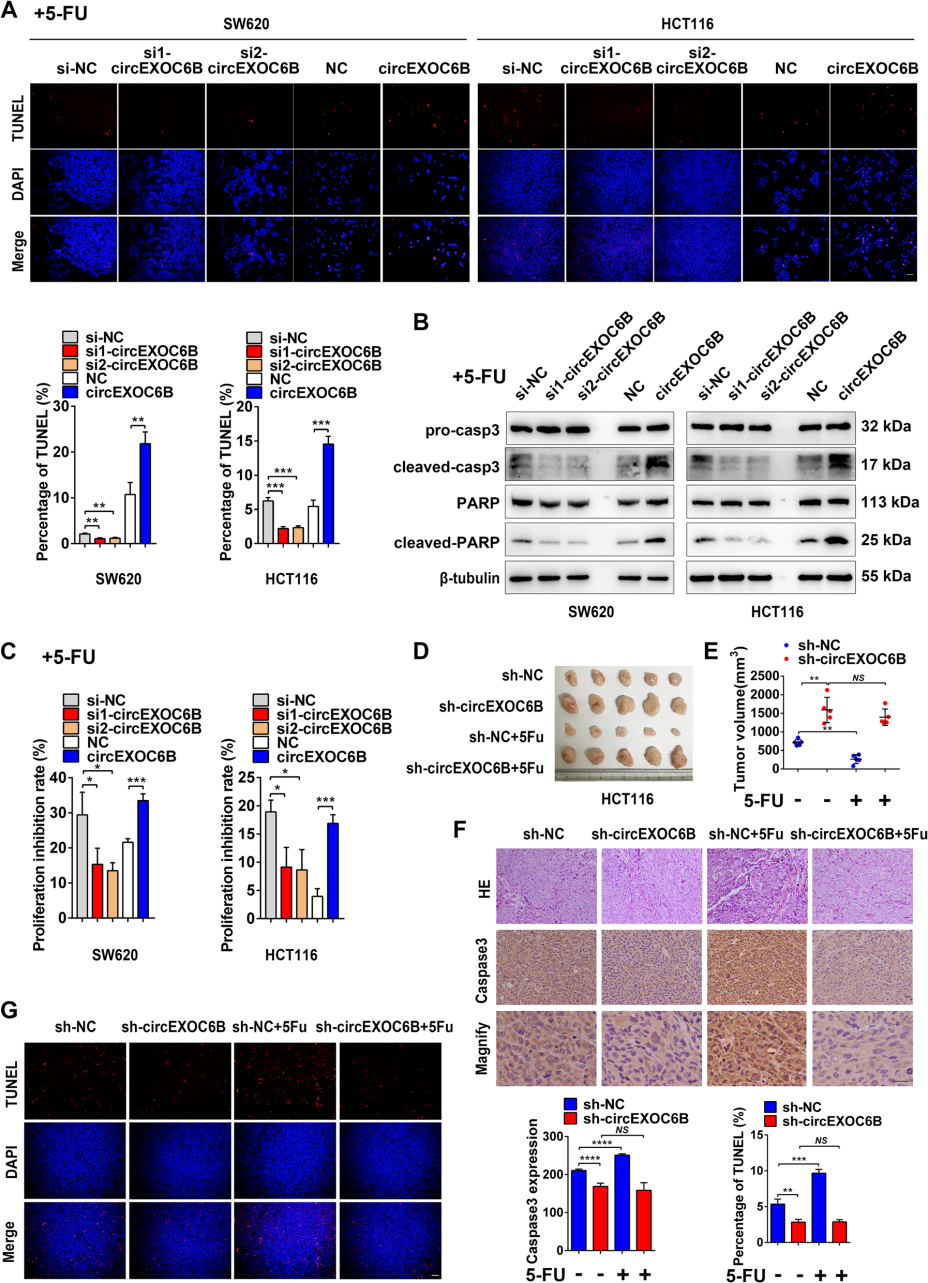
circEXOC6B enhances the sensitivity of CRC cells to 5-FU. **A** TUNEL showed that knockdown of circEXOC6B expression reduced the 5-FU-induced apoptosis of CRC cells, whereas overexpression of circEXOC6B made CRC cells more sensitive to 5-FU treatment. Scale: 50 μm. **B** Western blot results showed that knockdown or overexpression of circEXOC6B affected the expression of apoptotic markers in CRC cells after 5-FU treatment. **C** CCK8 revealed that downregulation of circEXOC6B antagonized the inhibitory role of 5-FU in CRC cell proliferation. In contrast, upregulation of circEXOC6B enhanced the effect of 5-FU on proliferation. **D** Gross image of subcutaneous tumors. **E** The tumor volume in the sh-circEXOC6B group showed no difference from the untreated group, whereas the tumor size was significantly decreased in the sh-NC group after 5-FU treatment. **F** IHC demonstrated that the apoptotic marker cleaved-caspase3 was not increased in the sh-circEXOC6B group but was dramatically elevated in the sh-NC group after 5-FU treatment. Scale: 20 μm. **G** TUNEL showed that the apoptosis in the sh-circEXOC6B group was unchanged with 5-FU treatment, whereas it was remarkably increased in the sh-NC group after 5-FU treatment. Scale: 50 μm

5-FU is widely used as a first-line chemotherapeutic drug for CRC. However, the effectiveness of 5-FU is reduced by intrinsic or acquired chemoresistance. Activation of the mTORC1 and HIF1A pathways has been considered to be the crucial reason for 5-FU resistance in recent years [19–22]. Our previous flow cytometry data in Fig. 2E indicated that circEXOC6B promoted the 5-FU-induced apoptosis of CRC cells. In addition, we found higher expression of circEXOC6B in the 5-FU-sensitive cell lines RKO, LoVo, HCT116, and HT-29 than in the 5-FU-nonsensitive cell line SW620 (Genomics of Drug Sensitivity in Cancer database: https://www.cancerrxgene.org/) (Fig. S9). Thus, circEXOC6B might enhance the sensitivity of CRC cells to 5-FU treatment by inhibiting the HIF1A-RRAGB-mTORC1 positive feedback loop. To further evaluate the effect of circEXOC6B on 5-FU treatment, TUNEL was performed to detect 5-FU-induced apoptosis. The results showed that lower circEXOC6B expression reduced the 5-FU-induced apoptosis of CRC cells, whereas higher circEXOC6B expression made CRC cells more sensitive to 5-FU treatment (Fig. 6A). Moreover, knockdown of circEXOC6B decreased the expression of the apoptotic markers cleaved caspase-3 and cleaved PARP while its overexpression increased their expression (Fig. 6B). Consistent with the apoptosis results, CCK8 assay revealed that downregulation of circEXOC6B antagonized the inhibitory role of 5-FU in CRC cell proliferation. In contrast, upregulation of circEXOC6B enhanced the effect of 5-FU on proliferation (Fig. 6C).

Furthermore, we constructed subcutaneous xenograft tumors to test the role of circEXOC6B in 5-FU treatment in vivo. After intraperitoneal injection of 5-FU, the tumor volume in the sh-circEXOC6B group showed no difference from the untreated group. In contrast, the tumor size was significantly decreased in the sh-NC group after 5-FU treatment (Fig. 6D and E). Additionally, the expression of the apoptotic marker cleaved caspase 3 was not increased in the sh-circEXOC6B group but was dramatically elevated in the sh-NC group (Fig. 6F). Consistent with the expression of cleaved caspase 3, TUNEL showed that the apoptosis in the sh-circEXOC6B group was unchanged with 5-FU treatment, whereas it was markedly increased in the sh-NC group after 5-FU treatment (Fig. 6G). These findings demonstrate that circEXOC6B enhances the sensitivity of CRC cells to 5-FU.

## Discussion

circRNA is a special type of transcript with a closed loop structure, which makes it more stable than other linear RNAs. In recent years, due to the development of high-throughput sequencing, the abnormal expression of circRNAs has been identified in a variety of cancers. Moreover, some of these circRNAs have been determined to act as oncogenes or suppressors that regulate cancer progression [23–25]. For instance, circGLIS3 promotes the proliferation of bladder cancer cells by adsorbing miR-1273f to upregulate cyclin D1 expression [26], circNDUFB2 inhibits cancer cell growth and metastasis and enhances anti-tumor immune responses by accelerating the ubiquitination of IGF2BP2 and recruiting immune cells into the tumor microenvironment in non-small cell lung cancer [27], the circRNAs CPM [28] and CRIM1 [29] promote chemoresistance in gastric cancer and nasopharyngeal carcinoma, respectively, and the circRNA Foxo3 inhibits chemoresistance in prostate cancer [30]. These findings support the role of circRNAs as promising therapeutic targets in cancer. However, the functions and mechanisms of aberrantly expressed circRNAs in CRC remain poorly understood.

In the present study, we discovered that circEXOC6B was downregulated in CRC and functioned as a novel suppressor of the mTORC1 pathway by binding to RRAGB. To the best of our knowledge, our research is the first to reveal the expression, function, and mechanism of circEXOC6B in CRC. Our results indicated that the downregulation of circEXOC6B was inversely related to tumor size, lymphatic metastasis, and TNM stage and associated with poor prognosis. In addition, gain- and loss-of-function assays confirmed that circEXOC6B inhibited CRC growth and enhanced CRC sensitivity to 5-FU treatment. Consistent with our findings, circEXOC6B is also downregulated in ovarian cancer [31] and endometrial cancer [32]. Furthermore, Wang et al. [33] and Zheng et al. [34] revealed that circEXOC6B suppresses the proliferation, invasion, and migration of ovarian cancer cells by sponging miR-421 and miR-376c-3p, respectively. However, as a distinct mechanism from serving as a “sponge” of miRNAs, we explored a novel role of circEXOC6B in which it could bind to protein in CRC.

Although most published articles reported that circRNAs acted as miRNA “sponges” in cancer [26, 35, 36], circRNAs can also interact with proteins to play major roles in tumorigenesis and progression [37, 38]. For example, Chen et al. reported that circACTN4 recruited YBX1 and enhanced the interaction of YAP1 and β-catenin to activate the Hippo and Wnt signaling pathways and thereby promote the proliferation and metastasis of tumor cells in intrahepatic cholangiocarcinoma [39]. Zheng et al. found that the PTEN-generated circRNA circPTEN1 could bind to Smad4 to disrupt its interaction with Smad2/3, which suppressed CRC invasion and metastasis [40]. In addition, the interaction with proteins was considered to be the main mechanism of circRNAs due to the finding that most circRNAs were present in the form of protein-binding complexes [41]. Nonetheless, novel mechanisms of circRNAs have rarely been explored. Using RNA pull-down and mass spectrometry, we determined that RRAGB protein was the binding target of circEXOC6B. RRAGB is a member of the Ras-GTPase family. It binds to RRAGC/D to form heterodimers, which is the precondition for the activation of the mTORC1 pathway [9–11]. However, little is known about the regulation of the heterodimers. In this study, we found that circEXOC6B hindered the formation of heterodimers by competitively binding to RRAGB and blocking the binding of RRAGC/D. Hence, circEXOC6B suppressed mTORC1 signaling and promoted HIF1A translation.

A hypoxic microenvironment is a hallmark of malignancy. Theoretically, hypoxia inhibits cancer growth. However, cancer overcomes the adverse influence of hypoxia on tumor growth partially by inducing HIF1A expression. HIF1A forms a complex with HIF1B and then enters the nucleus and binds to the HRE (A/GCGTG) in the promoter region of its target genes to upregulate their transcription [42]. These targets are involved in tumor growth, apoptosis, angiogenesis, invasion, and metastasis [43–46]. Here, triggered by the interesting finding that HIF1A and RRAGB have a positive correlation at the mRNA level in CRC cell lines and CRC tissues, we ultimately determined that RRAGB was one of the targets of HIF1A. RRAGB has been reported to be upregulated in CRC tissues and to have a negative correlation with patient prognosis [47]. Overall, we indicated the presence of a HIF1A-RRAGB-mTORC1 positive feedback loop in CRC that boosted tumor progression. However, circEXOC6B antagonized this loop by binding to RRAGB to inhibit CRC growth.

In addition, circEXOC6B has been reported to be able to enhance the sensitivity of tumor cells to paclitaxel in ovarian cancer [34]. Herein, we found that circEXOC6B promoted the apoptosis of CRC cells induced by 5-FU treatment in vitro and in vivo. 5-FU is widely used in the clinic to treat various cancers, including advanced CRC [48]. However, drug resistance significantly limits its anti-tumor effect [49]. As reported in numerous studies, the mTOR and HIF1A signaling pathways play crucial roles in chemotherapy resistance [50–53]. Kawakami et al. [19] determined that the mTOR pathway was excessively activated in 5-FU-resistant gastric cancer cells and that mTOR inhibitors could reduce the number of drug-resistant cells. Ha et al. [20] found that inhibition of the mTOR signaling pathway by a novel quinazolinone induced the apoptosis and autophagy of 5-FU-resistant CRC cells. As for HIF1A, Nakamura et al. [21] showed that a higher expression of HIF1A in gastric cancer was correlated with a poor response to 5-FU therapy and a shorter survival time of patients. In addition, Rohwer et al. [22] reported that HIF-1a governed chemoresistance in gastric cancer by modulating the p53 and NF-κB signaling pathways. In the present study, we verified that circEXOC6B could inhibit the HIF1A-RRAGB-mTORC1 positive feedback loop. Therefore, circEXOC6B may be a potential target for improving the susceptibility of CRC cells to 5-FU treatment.

## Conclusion

**Fig. 7:**
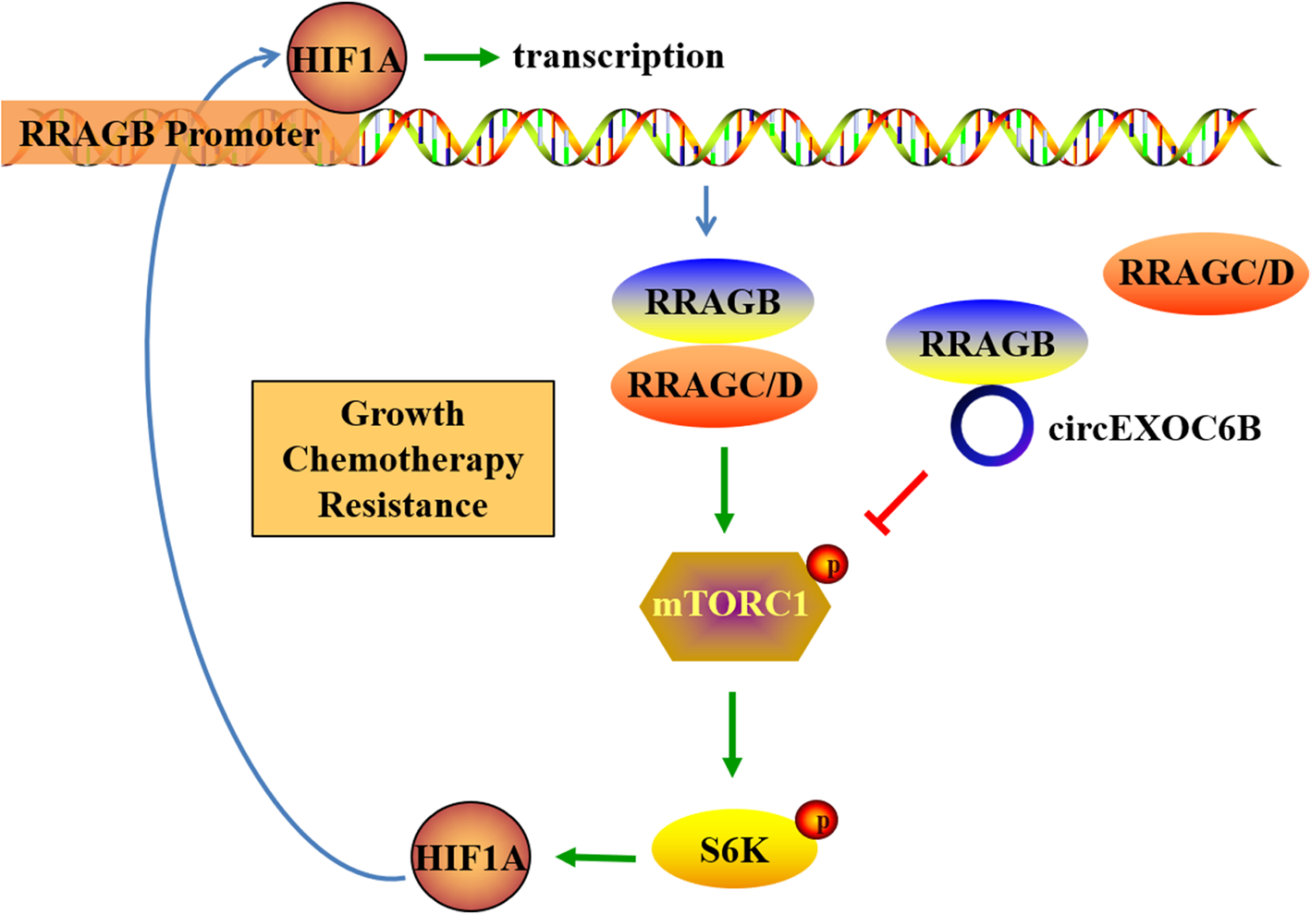
Illustration of circEXOC6B involvement in CRC

In summary, our findings revealed the existence of a HIF1A-RRAGB-mTORC1 positive feedback loop that drives CRC development. circEXOC6B can bind to the heterodimer formation site of RRAGB to disrupt its physical interaction with RRAGC/D, which suppresses HIF1A-RRAGB-mTORC1 positive feedback (Fig. 7). Thus, circEXOC6B inhibits CRC growth and increases the 5-FU-induced apoptosis of CRC. These results provide new insight into the crosstalk between the HIF1A and mTORC1 pathways and indicate a potential therapeutic target for blocking CRC progression.

## Supplementary Information

**Additional file 1: Supplementary Fig. S1.** Identification of circEXOC6B in CRC. (A) Heatmap showed the top five downregulated and five upregulated circRNAs in colon cancer screened by high-throughput sequencing (PMID25624062). (B) The consistent expression of circEXOC6B in GSE77661 and PMID25624062. (C) The schema showed that circEXOC6B is generated from the exon 3–6 of EXOC6B, and two pairs of divergent primers (1 and 2) were designed. PCR amplification with the Primer 2 followed by sanger sequencing confirmed circEXOC6B originated from the exon 3–6 of EXOC6B. The arrows showed the junctions between the exons of circEXOC6B. (D) PCR showed that circEXOC6B could only be amplified from cDNA in CRC cells using the divergent primer 2, and not from genomic DNA (gDNA). GAPDH as a control. (E) PCR indicated that circEXOC6B could resist RNase R digestion. GAPDH as a control. (F) CRC cells were treated with actinomycin D to inhibit new RNA generation, and RT-qPCR result revealed that circEXOC6B was more stable than GAPDH mRNA.

**Additional file 2: Supplementary Fig. S2.** Downregulation of circEXOC6B promoted the growth of RKO and Caco-2 cells. (A) The expression of circEXOC6B was knocked down by transfection with siRNA. (B) CCK8 was performed to detect the proliferation of RKO and Caco-2 cells after knockdown of circEXOC6B expression. (C) and (D) Colony formation assay showed that the downregulation of circEXOC6B promoted the clonogenicity of CRC cells. (E) Flow cytometry indicated that the decreased circEXOC6B accelerated cell cycle progression of CRC cells.

**Additional file 3: Supplementary Fig. S3.** Stable knockdown of circEXOC6B promoted the growth of CRC cells in vitro and in vivo. (A) The expression of circEXOC6B in SW620 and HCT116 cells with stable knockdown of circEXOC6B expression. (B) CCK8 and (C) Colony formation assays showed that the stable depletion of boosted the proliferation and clonogenicity of CRC cells compared with the control cells. (D) and (E) Subcutaneous tumor models demonstrated that the stable downregulation of circEXOC6B accelerated the growth of CRC cells in vivo. Scale: 20 μm.

**Additional file 4: Supplementary Fig. S4.** The subcellular distribution of circEXOC6B in CRC cell lines and CRC clinical sample. (A) PCR and (B) RT-qPCR were performed to detect the subcellular distribution of circEXOC6B in CRC cell lines. GAPDH and U6 were used as control. (C) FISH was performed to show the subcellular distribution of circEXOC6B in CRC clinical sample. Scale: 20 μm.

**Additional file 5: Supplementary Fig. S5.** RNA pull-down was performed in SW620 cells to identify the proteins interacting with circEXOC6B. The red frame marked RRAGB.

**Additional file 6: Supplementary Fig. S6.** The possible binding sites of RRAGB with circEXOC6B was predicted using catRAPID database.

**Additional file 7: Supplementary Fig. S7.** The positive correlation between HIF1A and RRAGB expression in CRC was validated using (A) GSE17538 (colon cancer, *n* = 238) and (B) The Cancer Genome Atlas (TCGA) (rectum cancer, *n* = 167) datasets.

**Additional file 8: Supplementary Fig. S8.** IHC showed that HIF1A, RRAGB and p-mTOR levels in CRC clinical samples were lower in the high circEXOC6B group (*n* = 11) than in the low circEXOC6B group (*n* = 11). Scale: 50 μm.

**Additional file 9: Supplementary Fig. S9.** The relative expression of circEXOC6B in LoVo, RKO, HCT116 and HT29 was higher than in SW620.

**Additional file 10: Supplementary Table S1.** Primer sequences are used in PCR or RT- qPCR.

**Additional file 11: Supplementary Table S2.** siRNAs are used for knockdown of circEXOC6B or HIF1A.

**Additional file 12: Supplementary Table S3.** The relationship between circEXOC6B expression and the clinicopathologic characteristics of CRC patients.

**Additional file 13: Supplementary Table S4.** Proteins might bind to circEXOC6B were identified by mass spectrometry (MS).

## Data Availability

All data generated or analyzed during this study are included in this published article and its Additional files.

## Abbreviations

CircRNA: Circular RNA
CRC: Colorectal cancer
Co-IP: Coimmunoprecipitation
ChIP: Chromatin immunoprecipitation
EXOC6B: Exocyst complex component 6B
FISH: Fluorescence in situ hybridization
gDNA: Genomic DNA
HR: Hazard ratio
HIF1A: Hypoxia inducible factor 1alpha
HRE: Hypoxia response element
IHC: Immunohistochemistry
IF: Immunofluorescence
miRNA: microRNA
mTORC1: Mammalian target of rapamycin complex 1
MS: Mass spectrometry
OS: Overall survival
RIP: RNA binding protein immunoprecipitation
RRAGB: Ras-related GTP binding B
RT-qPCR: Real-time quantitative polymerase chain reaction
siRNA: Small interfering RNA
S6K: Ribosomal protein S6 kinase B1
4E-BP1: 4E binding protein 1
5-FU: 5-Fluorouracil
