# Supplementary figures and images for: circEXOC6B interacting with RRAGB, an mTORC1 activator, inhibits the progression of colorectal cancer by antagonizing the HIF1A-RRAGB-mTORC1 positive feedback loop
Source: Mol Cancer. 2022 Jun 23;21:135. doi: 10.1186/s12943-022-01600-1 (PMC9219196; doi:10.1186/s12943-022-01600-1)

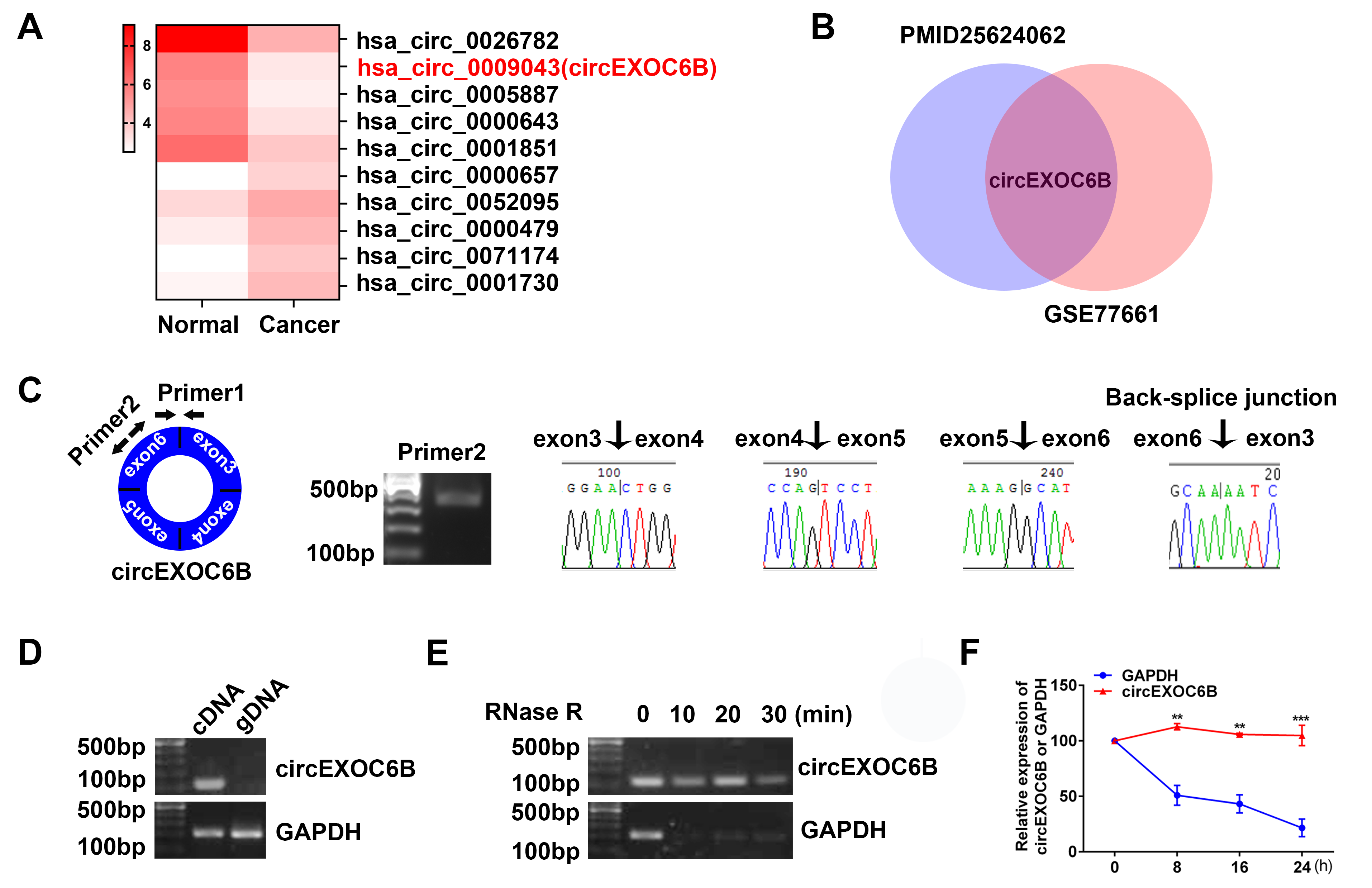

Supplement: Supplementary file 1 — Additional file 1: Supplementary Fig. S1. Identification of circEXOC6B in CRC. (A) Heatmap showed the top five downregulated and five upregulated circRNAs in colon cancer screened by high-throughput sequencing (PMID25624062). (B) The consistent expression of circEXOC6B in GSE77661 and PMID25624062. (C) The schema showed that circEXOC6B is generated from the exon 3–6 of EXOC6B, and two pairs of divergent primers (1 and 2) were designed. PCR amplification with the Primer 2 followed by sanger sequencing confirmed circEXOC6B originated from the exon 3–6 of EXOC6B. The arrows showed the junctions between the exons of circEXOC6B. (D) PCR showed that circEXOC6B could only be amplified from cDNA in CRC cells using the divergent primer 2, and not from genomic DNA (gDNA). GAPDH as a control. (E) PCR indicated that circEXOC6B could resist RNase R digestion. GAPDH as a control. (F) CRC cells were treated with actinomycin D to inhibit new RNA generation, and RT-qPCR result revealed that circEXOC6B was more stable than GAPDH mRNA. [file 12943_2022_1600_MOESM1_ESM.tif]

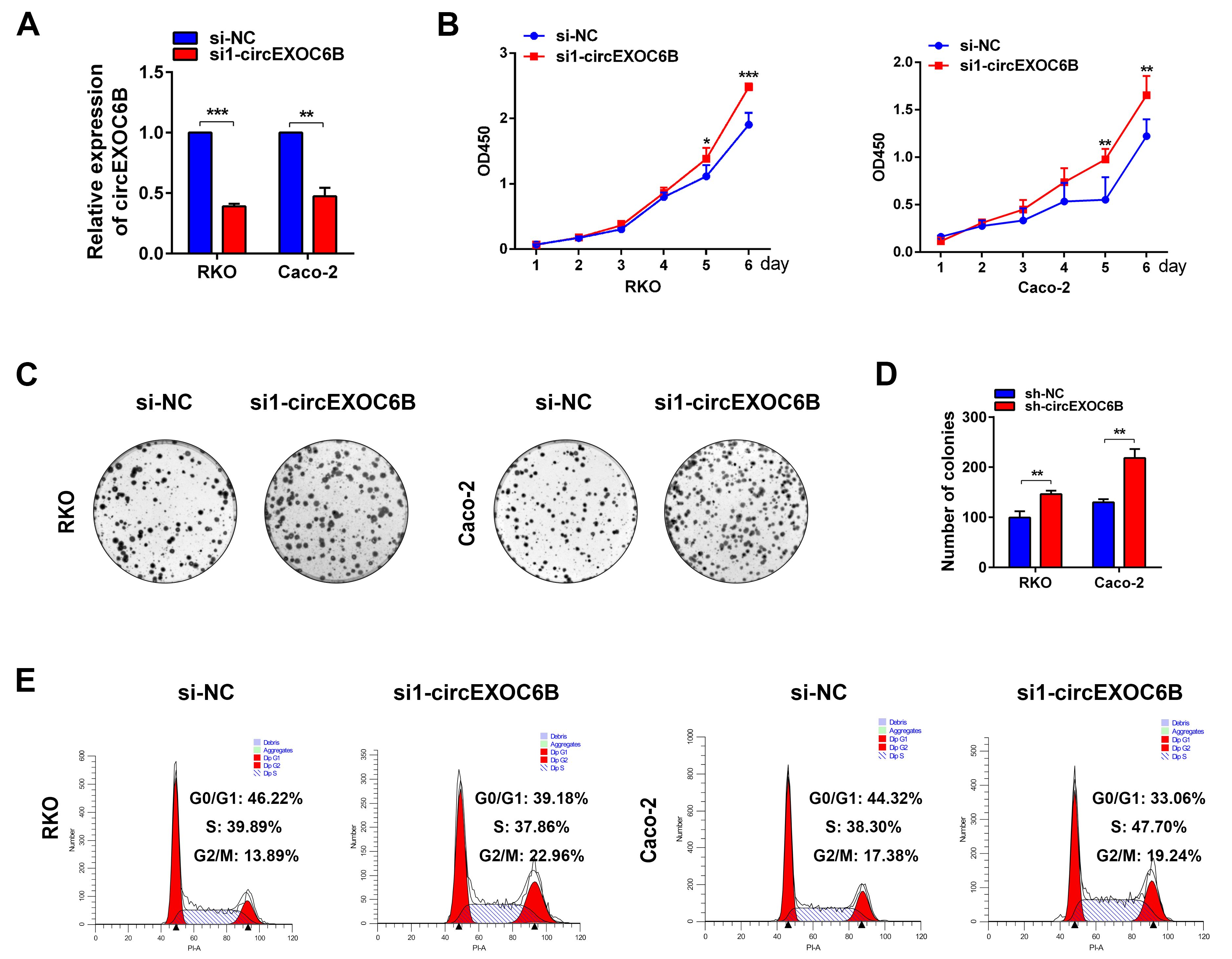

Supplement: Supplementary file 2 — Additional file 2: Supplementary Fig. S2. Downregulation of circEXOC6B promoted the growth of RKO and Caco-2 cells. (A) The expression of circEXOC6B was knocked down by transfection with siRNA. (B) CCK8 was performed to detect the proliferation of RKO and Caco-2 cells after knockdown of circEXOC6B expression. (C) and (D) Colony formation assay showed that the downregulation of circEXOC6B promoted the clonogenicity of CRC cells. (E) Flow cytometry indicated that the decreased circEXOC6B accelerated cell cycle progression of CRC cells. [file 12943_2022_1600_MOESM2_ESM.tif]

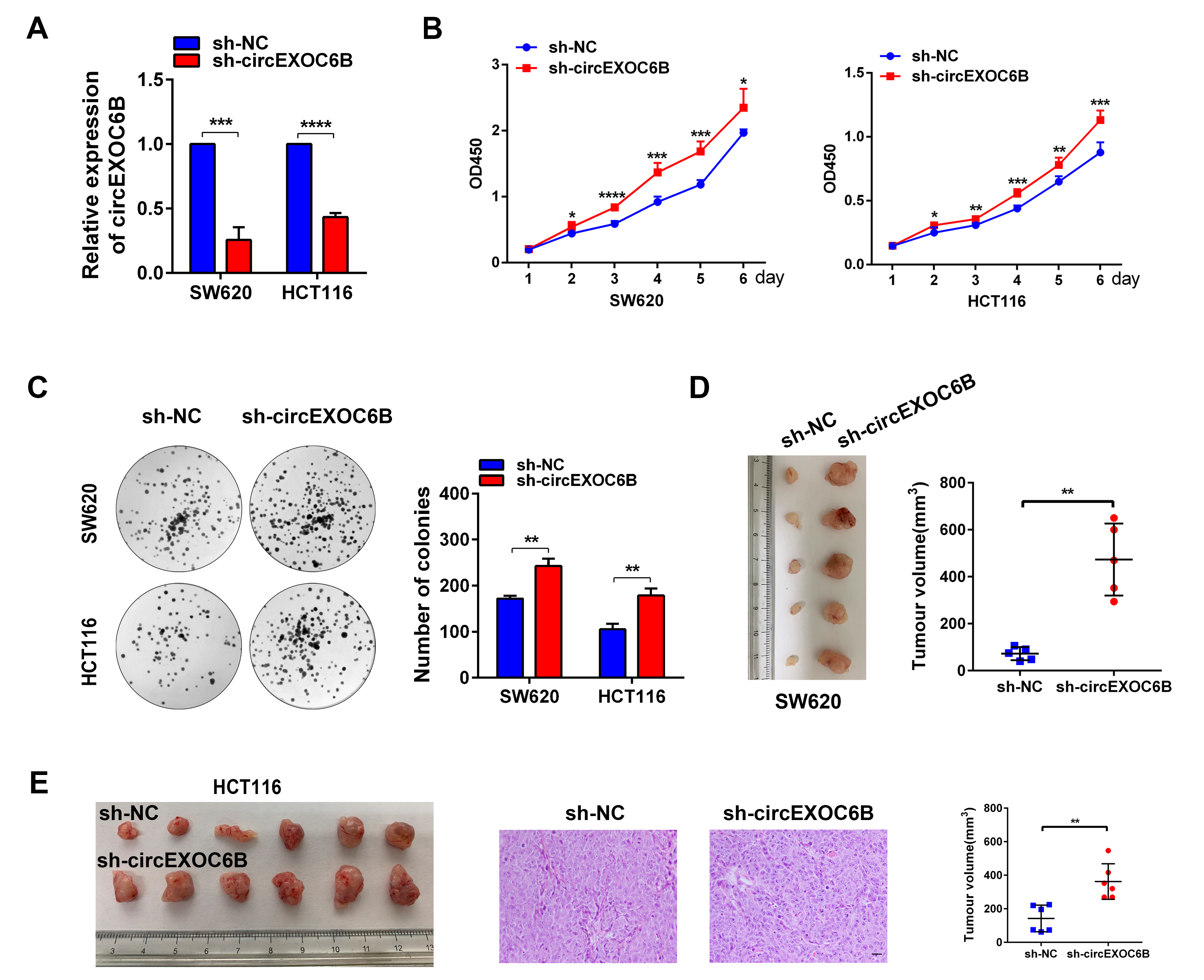

Supplement: Supplementary file 3 — Additional file 3: Supplementary Fig. S3. Stable knockdown of circEXOC6B promoted the growth of CRC cells in vitro and in vivo. (A) The expression of circEXOC6B in SW620 and HCT116 cells with stable knockdown of circEXOC6B expression. (B) CCK8 and (C) Colony formation assays showed that the stable depletion of boosted the proliferation and clonogenicity of CRC cells compared with the control cells. (D) and (E) Subcutaneous tumor models demonstrated that the stable downregulation of circEXOC6B accelerated the growth of CRC cells in vivo. Scale: 20 μm. [file 12943_2022_1600_MOESM3_ESM.tif]

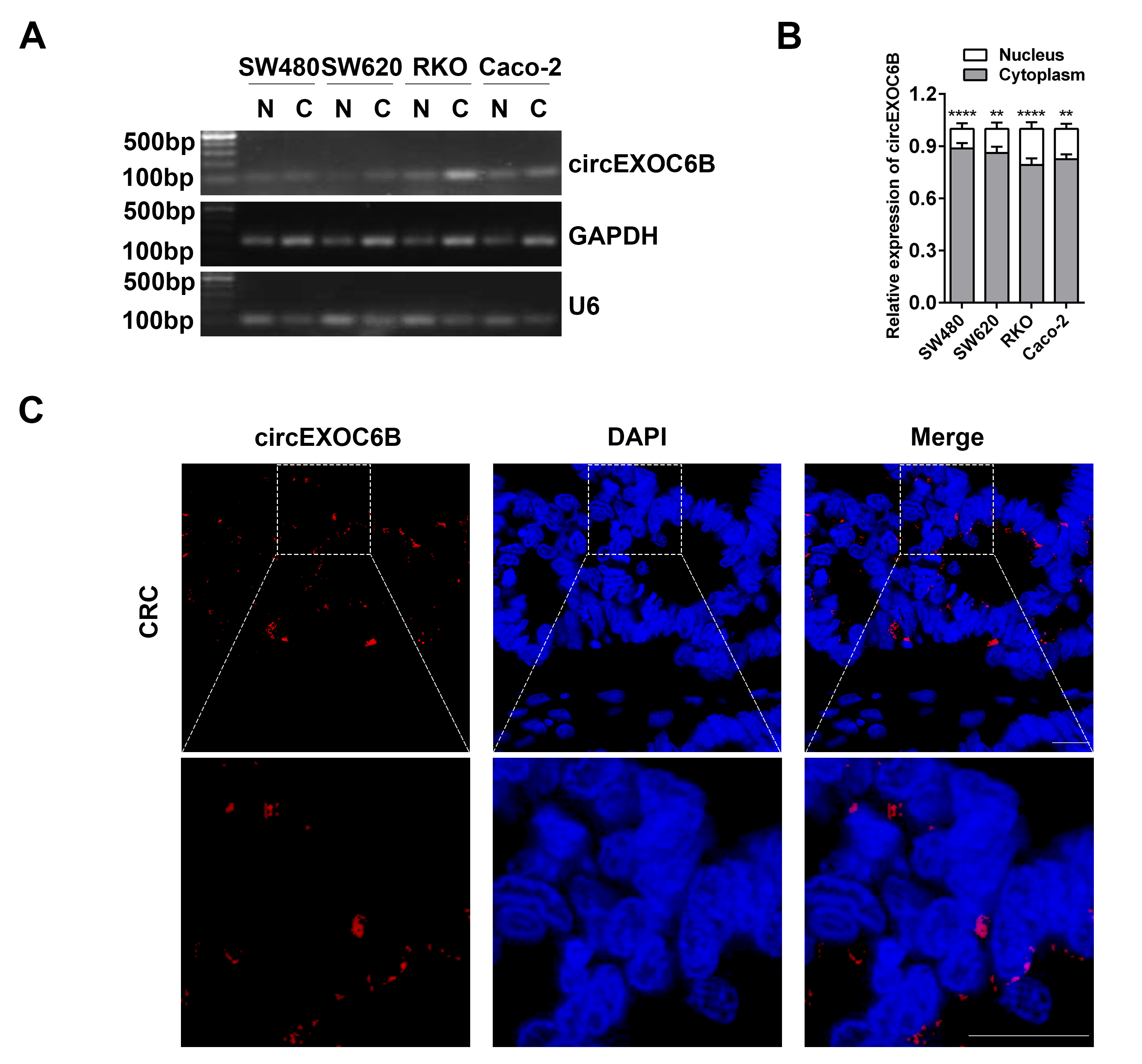

Supplement: Supplementary file 4 — Additional file 4: Supplementary Fig. S4. The subcellular distribution of circEXOC6B in CRC cell lines and CRC clinical sample. (A) PCR and (B) RT-qPCR were performed to detect the subcellular distribution of circEXOC6B in CRC cell lines. GAPDH and U6 were used as control. (C) FISH was performed to show the subcellular distribution of circEXOC6B in CRC clinical sample. Scale: 20 μm. [file 12943_2022_1600_MOESM4_ESM.tif]

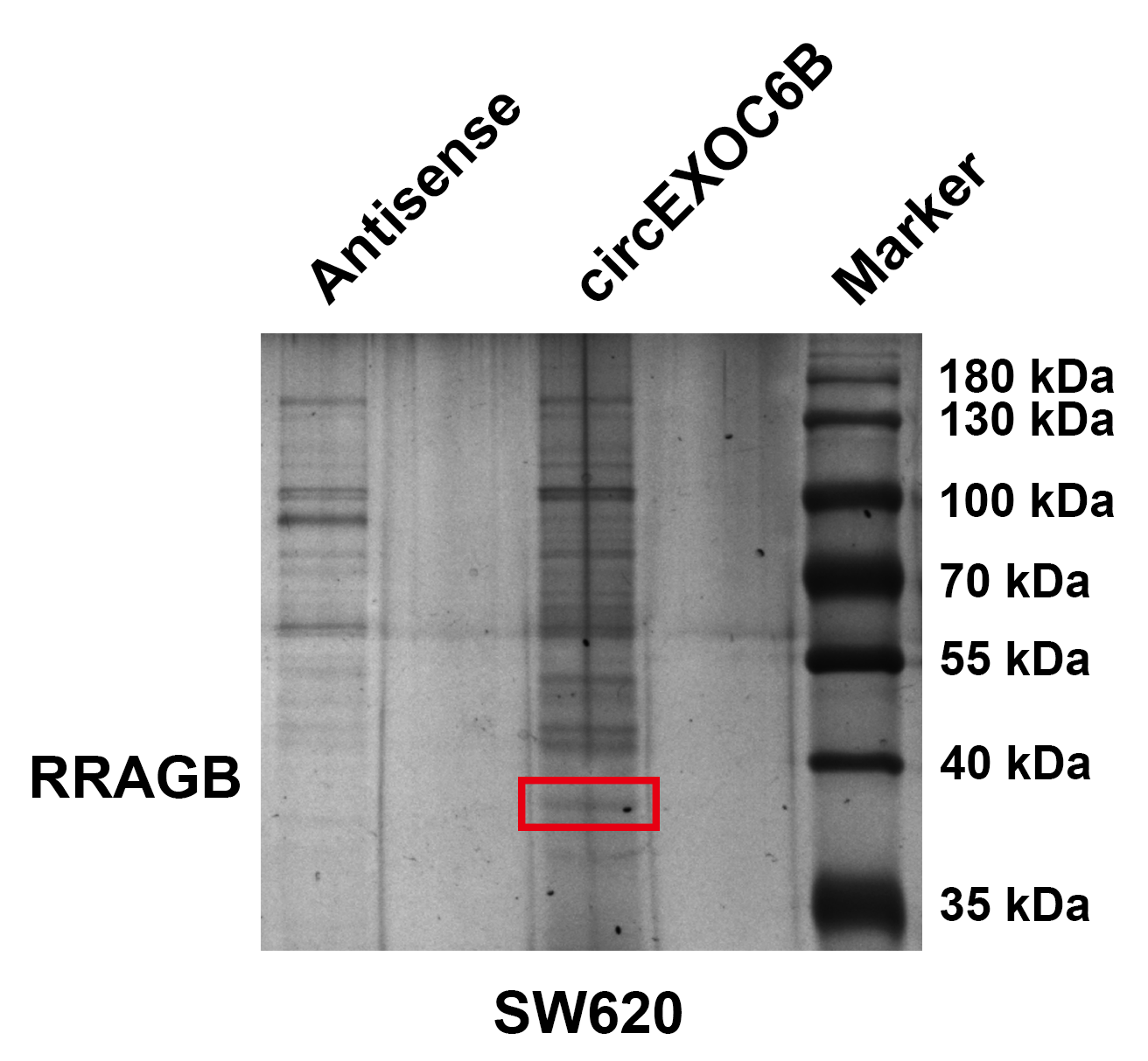

Supplement: Supplementary file 5 — Additional file 5: Supplementary Fig. S5. RNA pull-down was performed in SW620 cells to identify the proteins interacting with circEXOC6B. The red frame marked RRAGB. [file 12943_2022_1600_MOESM5_ESM.tif]

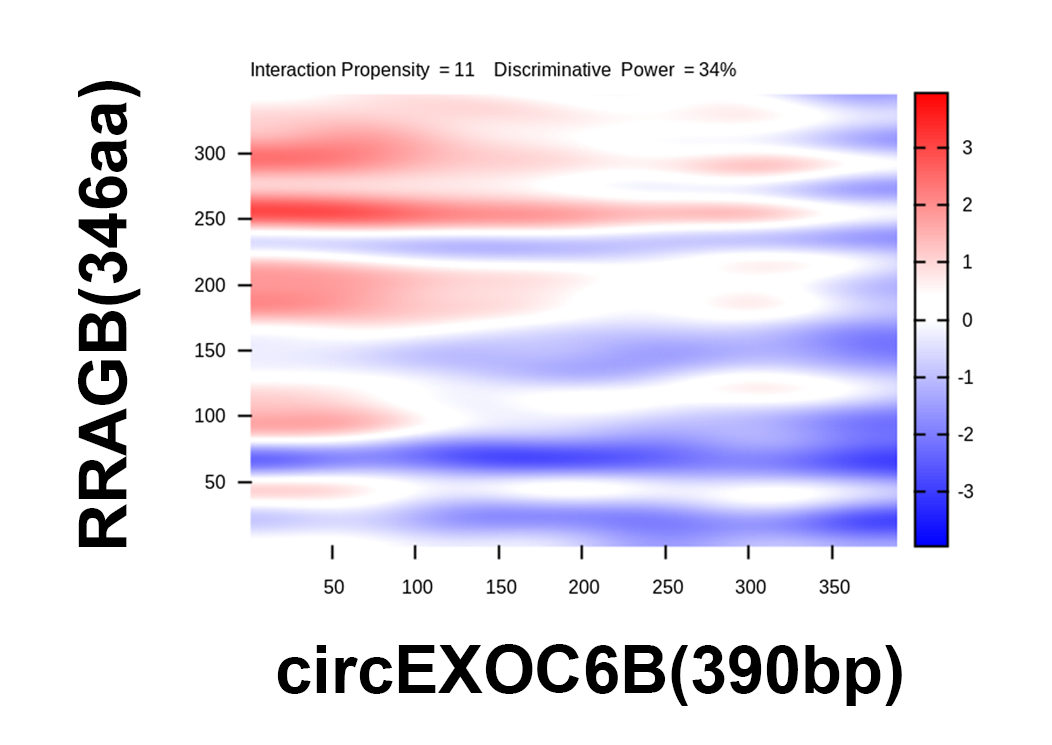

Supplement: Supplementary file 6 — Additional file 6: Supplementary Fig. S6. The possible binding sites of RRAGB with circEXOC6B was predicted using catRAPID database. [file 12943_2022_1600_MOESM6_ESM.tif]

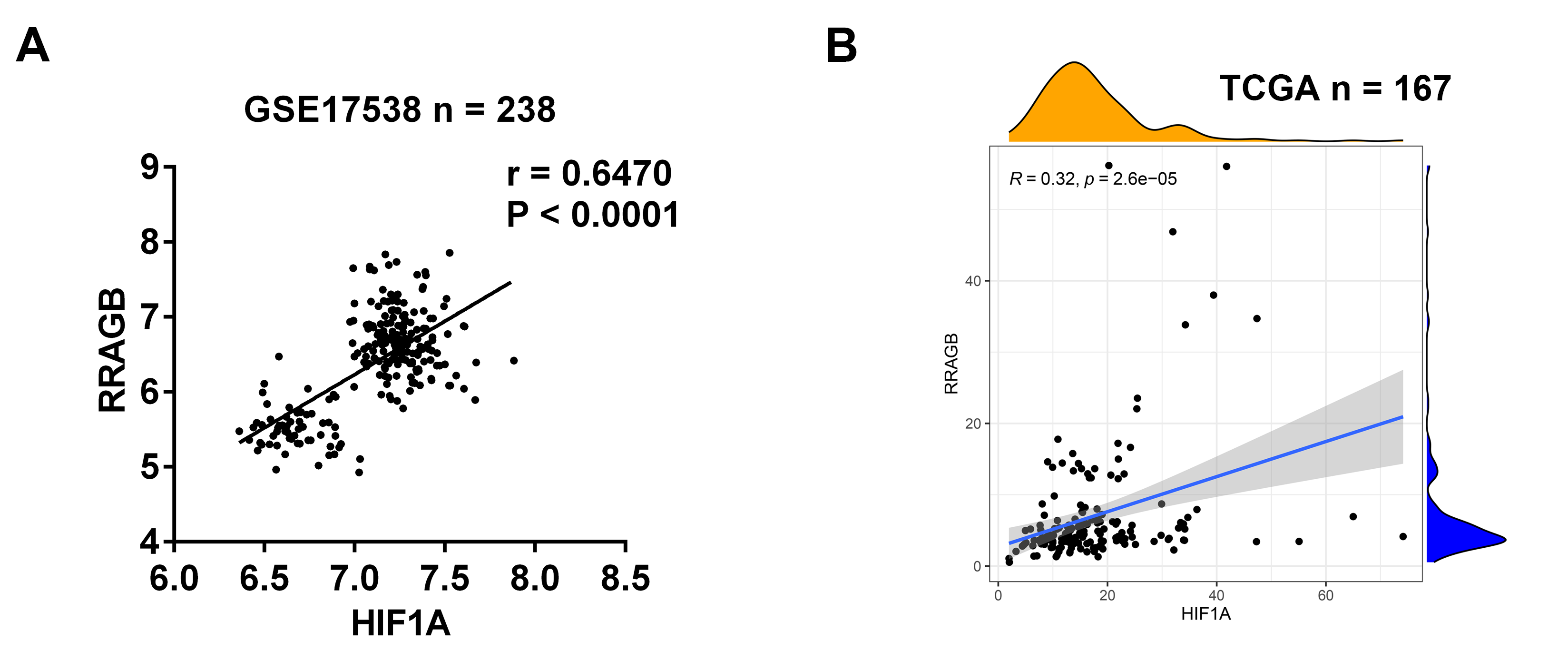

Supplement: Supplementary file 7 — Additional file 7: Supplementary Fig. S7. The positive correlation between HIF1A and RRAGB expression in CRC was validated using (A) GSE17538 (colon cancer, n = 238) and (B) The Cancer Genome Atlas (TCGA) (rectum cancer, n = 167) datasets. [file 12943_2022_1600_MOESM7_ESM.tif]

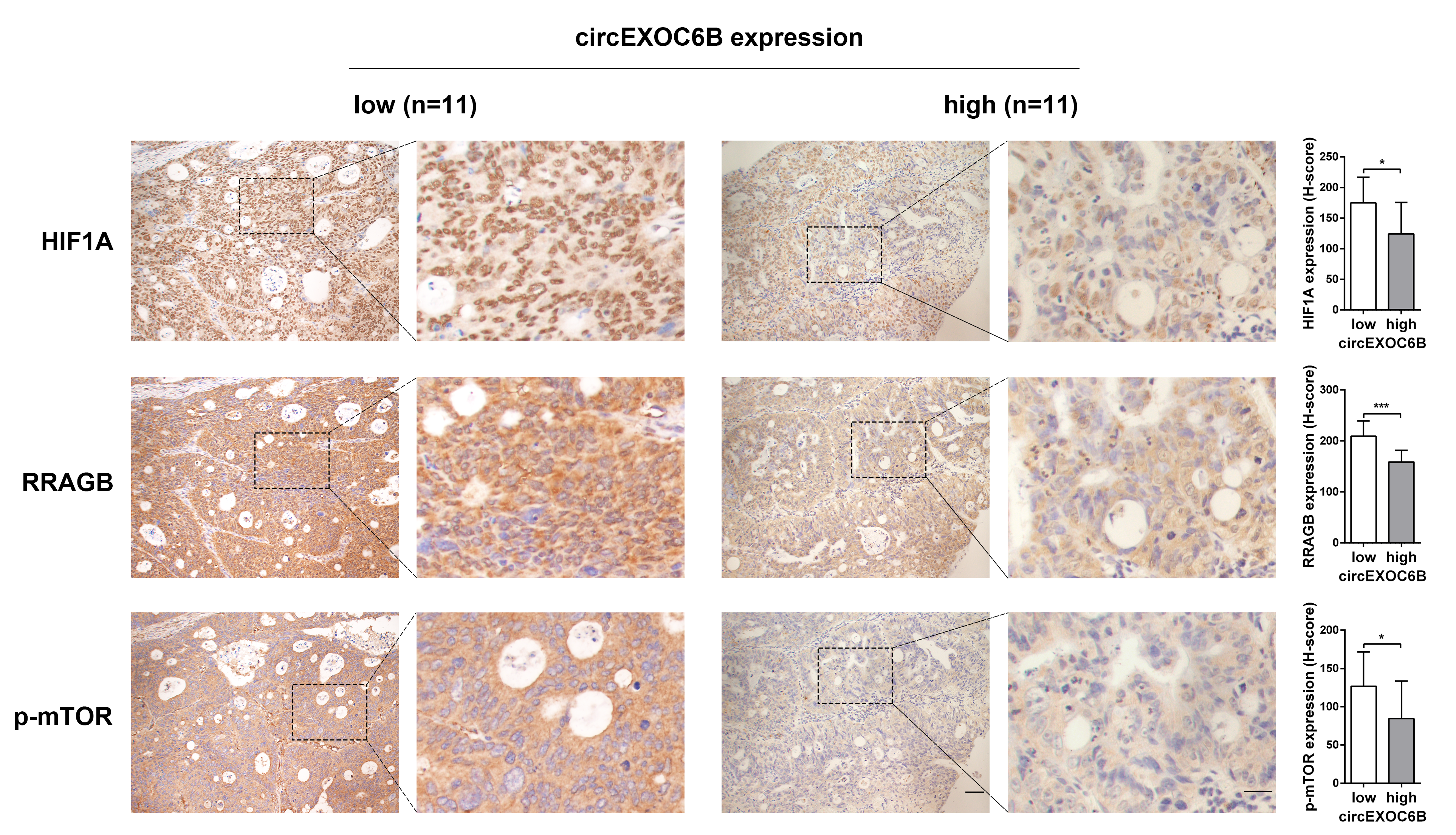

Supplement: Supplementary file 8 — Additional file 8: Supplementary Fig. S8. IHC showed that HIF1A, RRAGB and p-mTOR levels in CRC clinical samples were lower in the high circEXOC6B group (n = 11) than in the low circEXOC6B group (n = 11). Scale: 50 μm. [file 12943_2022_1600_MOESM8_ESM.tif]
