# Supplementary material for: circEXOC6B interacting with RRAGB, an mTORC1 activator, inhibits the progression of colorectal cancer by antagonizing the HIF1A-RRAGB-mTORC1 positive feedback loop
Source: Mol Cancer. 2022 Jun 23;21:135. doi: 10.1186/s12943-022-01600-1 (PMC9219196; doi:10.1186/s12943-022-01600-1)
Supplement: Supplementary file 10 — Additional file 10: Supplementary Table S1. Primer sequences are used in PCR or RT- qPCR. [file 12943_2022_1600_MOESM10_ESM.doc]

**Supplementary Table S1.** **Primer sequences are used in PCR or RT- qPCR**

|  | | Forward primer (5’-3’) | Reverse primer (5’-3’) | |
| --- | --- | --- | --- | --- |
| circEXOC6B-Primer1 | ACTGCCATGAAGCAAAATCAAGT | | GTCGACACTGCTTCAGCTCT |  |
| circEXOC6B-Primer2 | GCATCCGCAAACATTCAG | | GTTGTCCACCATCACCTT |  |
| HIF1A | CACCACAGGACAGTACAGGAT | | CGTGCTGAATAATACCACTCACA |  |
| RRAGB | AACCGGAAGGATCGCTTGG | | CGACGTGTGTCTCTGGCAA |  |
| RRAGB-Promoter-P1 | ATTTCCTCAGTTACAGTT | | CTACATGTATTATTTACACG |  |
| RRAGB-Promoter-P2 | CCTTTGGTAGCGGCAGTC | | TCAGGAACCCAGGTATCTCA |  |
| RRAGB-Promoter-P3 | TACTTTGTACCACGGAATCACT | | CTCAACTGCCTATTCTCATCG |  |
| 18sRNA | ACACGGACAGGATTGACAGA | | GGACATCTAAGGGCATCACA |  |
| GAPDH | GGAGCGAGATCCCTCCAAAAT | | GGCTGTTGTCATACTTCTCATGG |  |
| U6 | CTCGCTTCGGCAGCACA | | AACGCTTCACGAATTTGCGT |  |
| β-actin | GAAGAGCTACGAGCTGCCTGA | | CAGACAGCACTGTGTTGGCG |  |
