# Supplementary material for: circEXOC6B interacting with RRAGB, an mTORC1 activator, inhibits the progression of colorectal cancer by antagonizing the HIF1A-RRAGB-mTORC1 positive feedback loop
Source: Mol Cancer. 2022 Jun 23;21:135. doi: 10.1186/s12943-022-01600-1 (PMC9219196; doi:10.1186/s12943-022-01600-1)
Supplement: Supplementary file 11 — Additional file 11: Supplementary Table S2. siRNAs are used for knockdown of circEXOC6B or HIF1A. [file 12943_2022_1600_MOESM11_ESM.doc]

**Supplementary Table S2.** **siRNAs are used for knockdown of circEXOC6B or HIF1A**

|  | Target sequences (5’-3’) |
| --- | --- |
| circEXOC6B siRNA-1 | CCATGAAGCAAAATCAAGT |
| circEXOC6B siRNA-2 | CTGCCATGAAGCAAAATCA |
| HIF1A siRNA-1 | GCCACATCATCACCATATA |
| HIF1A siRNA-2 | GGAACATGATGGTTCACTT |
