# Supplementary material for: circEXOC6B interacting with RRAGB, an mTORC1 activator, inhibits the progression of colorectal cancer by antagonizing the HIF1A-RRAGB-mTORC1 positive feedback loop
Source: Mol Cancer. 2022 Jun 23;21:135. doi: 10.1186/s12943-022-01600-1 (PMC9219196; doi:10.1186/s12943-022-01600-1)
Supplement: Supplementary file 12 — Additional file 12: Supplementary Table S3. The relationship between circEXOC6B expression and the clinicopathologic characteristics of CRC patients. [file 12943_2022_1600_MOESM12_ESM.doc]

**Supplementary Table S3. The relationship between circEXOC6B expression and the clinicopathologic characteristics of CRC patients**

| Characteristics | circEXOC6B expression (n = 78) ^a^ | |  |
| --- | --- | --- | --- |
|  | Low (n = 39) | High (n = 39) | P value |
| Age (years) ^b^ |  |  |  |
| < 60 | 19 | 18 | 0.8206 |
| >= 60 | 20 | 21 |  |
| Gender |  |  |  |
| Male | 21 | 22 | 0.8199 |
| Female | 18 | 17 |  |
| Cancer location |  |  |  |
| Right colon | 11 | 9 | 0.3732 |
| Left colon | 16 | 12 |  |
| Rectum | 12 | 18 |  |
| Diameter (cm) ^c^ |  |  |  |
| < 4.5 | 13 | 25 | **0.0066** |
| >= 4.5 | 26 | 14 |  |
| Differentiation |  |  |  |
| Well | 4 | 7 | 0.2587 |
| Moderate | 33 | 27 |  |
| Poor | 2 | 5 |  |
| Serosal Invasion |  |  |  |
| No | 8 | 13 | 0.2018 |
| Yes | 31 | 26 |  |
| Lymphatic metastasis |  |  |  |
| No | 13 | 22 | **0.0405** |
| Yes | 26 | 17 |  |
| Distant metastasis |  |  |  |
| No | 32 | 31 | 0.7739 |
| Yes | 7 | 8 |  |
| TNM classification |  |  |  |
| I-II | 12 | 24 | **0.0064** |
| III-IV | 27 | 15 |  |

a) Patients were dichotomized based on the median expression level of circEXOC6B.

b) Group of age was performed according to median.

c) Tumor size was grouped according to median.
